# Inference of phylogenetic trees directly from raw sequencing reads using Read2Tree

**DOI:** 10.1038/s41587-023-01753-4

**Published:** 2023-04-20

**Authors:** David Dylus, Adrian Altenhoff, Sina Majidian, Fritz J. Sedlazeck, Christophe Dessimoz

**Affiliations:** 1 Department of Computational Biology, University of Lausanne, Lausanne, Switzerland; 2 SIB Swiss Institute of Bioinformatics, Lausanne, Switzerland; 3 Department of Computer Science, ETH, Zurich, Switzerland; 4 Human Genome Sequencing Center, Baylor College of Medicine, Houston, TX USA; 5 Department of Computer Science, Rice University, Houston, TX USA; 6 Department of Computer Science, University College London, London, UK; 7 Centre for Life’s Origins and Evolution, Department of Genetics, Evolution and Environment, University College London, London, UK; 8 Present Address: F. Hoffmann-La Roche Ltd, Immunology, Infectious Disease, and Ophthalmology (I2O), Roche Pharmaceutical Research and Early Development (pRED), Basel, Switzerland

**Keywords:** Phylogeny, Genome informatics, Phylogenetics, Comparative genomics

## Abstract

Current methods for inference of phylogenetic trees require running complex pipelines at substantial computational and labor costs, with additional constraints in sequencing coverage, assembly and annotation quality, especially for large datasets. To overcome these challenges, we present Read2Tree, which directly processes raw sequencing reads into groups of corresponding genes and bypasses traditional steps in phylogeny inference, such as genome assembly, annotation and all-versus-all sequence comparisons, while retaining accuracy. In a benchmark encompassing a broad variety of datasets, Read2Tree is 10–100 times faster than assembly-based approaches and in most cases more accurate—the exception being when sequencing coverage is high and reference species very distant. Here, to illustrate the broad applicability of the tool, we reconstruct a yeast tree of life of 435 species spanning 590 million years of evolution. We also apply Read2Tree to >10,000 *Coronaviridae* samples, accurately classifying highly diverse animal samples and near-identical severe acute respiratory syndrome coronavirus 2 sequences on a single tree. The speed, accuracy and versatility of Read2Tree enable comparative genomics at scale.

## Abstract (web summary)

Phylogenetic trees are generated from sequencing reads without genome assembly or annotation.

## Main

Phylogenetic trees depict evolutionary relationships among biological entities. These entities can be species—as in the tree of life^1–4^. They can also be cancerous cells in tumor progression trees^5^ or developmental lineage trees^6^, viral and bacterial strains in infectious outbreaks^7^, cells, or genes in trees used to propagate molecular function annotations among model and nonmodel species^8,9^. Owing to this pervasiveness, methods to infer phylogenetic trees are among the most used and cited software tools in all of life sciences.

In the context of species tree inference, the availability of genome-wide sequencing has made it routine to consider as many marker genes per taxon as the genomes provide. This ‘phylogenomic’ approach has resolved many key aspects of the eukaryotic tree of life, such as the relation among deep angiosperm clades^10^, the position of sea squirts within chordates^11^, the Ecdysozoa clade^12^, the Lophotrochozoa clade^13^ and relations among main myriapod clades^14^, among many others.

**Fig. 1:**
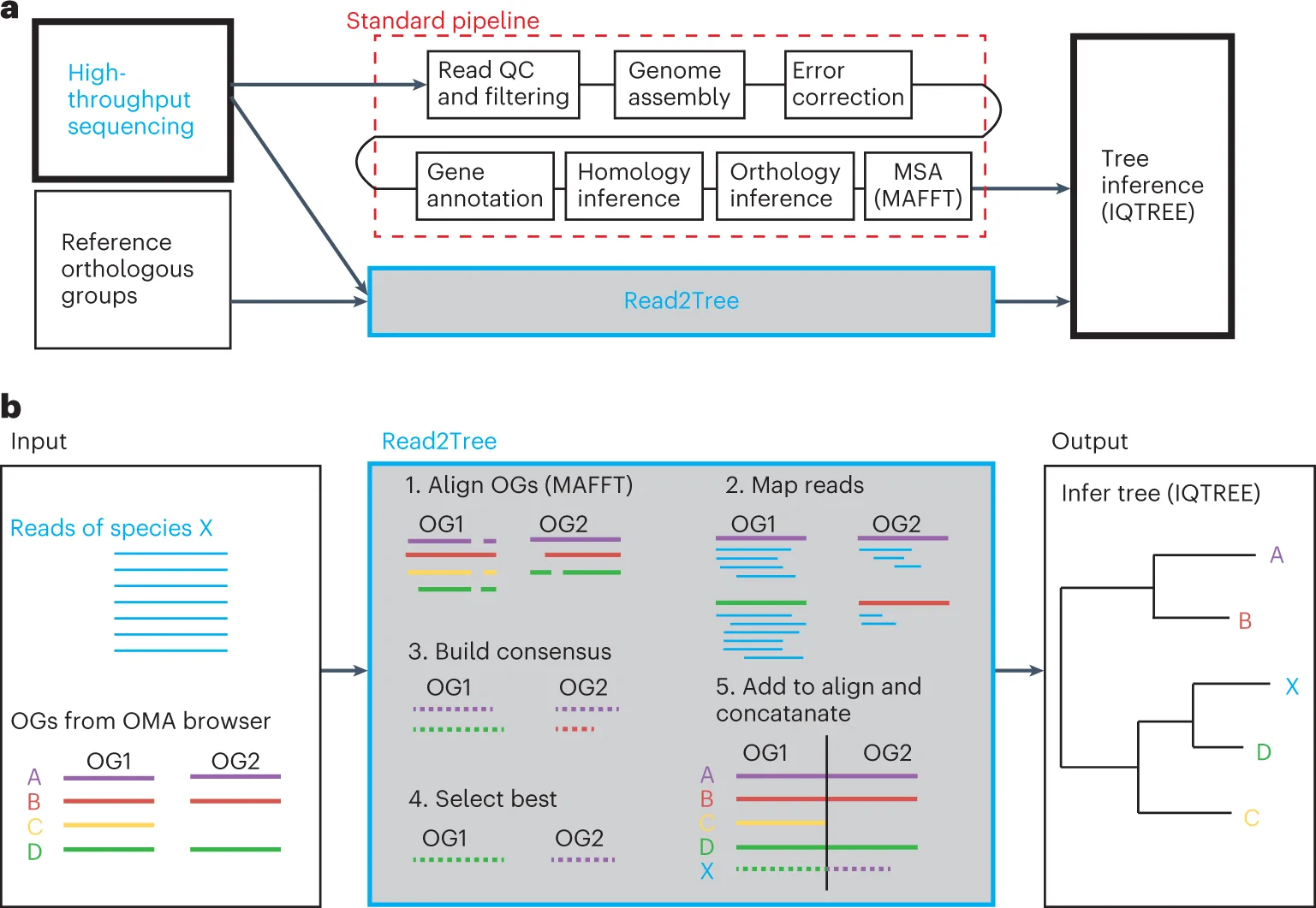
Strategy and pipeline explanation. **a**, Read2Tree aims at side stepping many time-intensive and costly pipeline steps to obtain a phylogenetic tree when using many species, therefore going from read to tree. **b**, Overview of the Read2Tree pipeline.

Nevertheless, despite rapid improvements in the quality and cost of sequencing^15,16^, the data analysis required to infer phylogenetic trees remains extremely laborious and computationally intensive^17^. Phylogenomic studies require multiple costly steps, each of which can be major research endeavors (Fig. 1): the curation of raw reads, the de novo assembly often including multiple rounds of error corrections and scaffolding either with one or multiple technologies^18^, the annotation and characterization of important genes, the identification and comparison of orthologous genes, and the tree inference from orthologous markers. The current best practices optimize this process with combinations of technologies, such as long- and short-read sequencing, and multiple rounds of parameter optimizations across multiple pipelines.

The current trend is to sequence ever more species and samples. The Earth BioGenome Project, launched in November 2018, aims at sequencing ‘all 1.5 million known animal, plant, protozoan and fungal species on Earth’ within the coming decade^19^. The constituting consortia are making progress streamlining and optimizing the sequencing and annotation process, but the orthology inference and tree inference steps remain highly challenging. In parallel, considerable genome sequencing activity is taking place in individual laboratories, with sample sizes of hundreds to thousands of genomes per study becoming common^16^. However, depending on the species of interest, high-quality reference genomes are often lacking, and individual laboratories often lack the computational infrastructure or expertise to fully leverage the data across individual analysis steps. This is exemplified in major consortia-led studies requiring years and millions of dollars to elucidate the evolution of certain species of interest or, most recently, the use of various pipelines to assess variation and report assemblies from severe acute respiratory syndrome coronavirus 2 (SARS-CoV-2). Thus, a major bottleneck is becoming the harmonized analysis of these large-scale datasets to avoid certain biases or artifacts.

In this Article, we introduce Read2Tree, an approach to infer species trees, which works by directly processing raw sequencing reads into groups of corresponding genes—bypassing genome assembly, annotation or all-versus-all sequence comparisons. Read2Tree is able to provide a full phylogenetic comparison of hundreds of samples in a fraction of time compared with current established pipelines. Crucially, the speedup is achieved without compromising the accuracy of the resulting trees. In addition, Read2Tree is able to also provide accurate trees and species comparisons using only low-coverage (0.1×) datasets as well as RNA versus genomic sequencing and operates on long or short reads. This makes Read2Tree a highly versatile method to obtain key insights from a single sample, scaling up to thousands of samples. To establish this approach, we assess its performance on a battery of genomic and transcriptomic datasets spanning different kingdoms, divergence time and sequencing technology. Subsequently, we apply Read2Tree to construct a large yeast tree of life and apply it to compare SARS-CoV-2 samples—thus highlighting the accuracy (for example, compared with National Center for Biotechnology Information (NCBI) classification) and speed of Read2Tree.

## Results

**Fig. 2:**
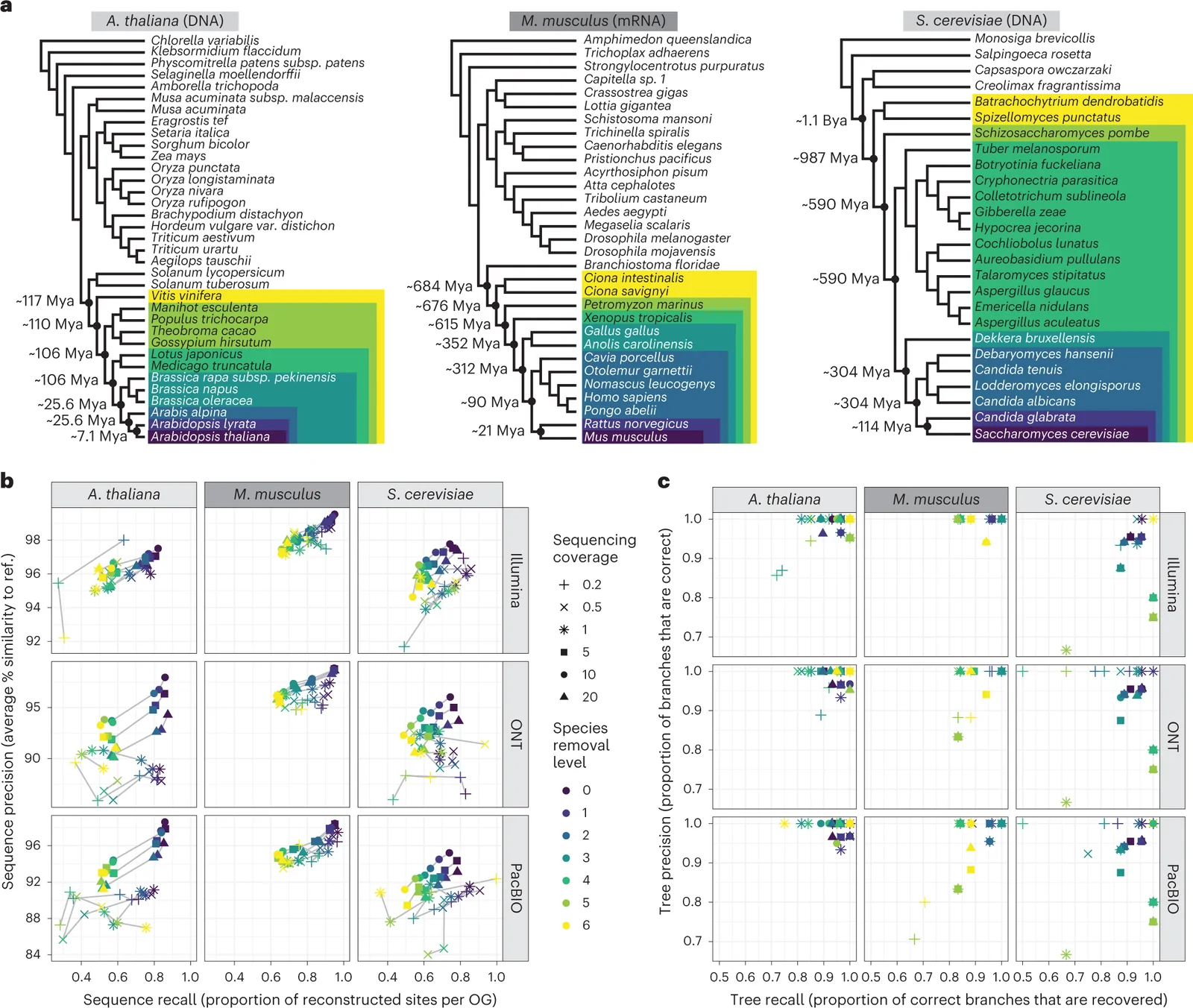
Benchmark of Read2Tree using three different datasets, six different coverage levels and three sequencing technologies. **a**, Phylogenetic trees of reference datasets. In dark purple (bottom) are the species used for mapping. The colors represent species removal to assess the dependency on closest neighbors in the reference datasets. Timepoints were obtained from timetree.org^59^. **b**, Read2Tree sequences are more similar (percentage identity) and more complete with increasing coverage and decreasing distance to a more closely related species. The best sequence identity is obtained for Illumina data. The colors convey the increasing evolutionary distance to the closest reference species (ref.). **c**, The precision and recall of trees reconstructed using Read2Tree after collapsing branches below 90% support.

**Fig. 3:**
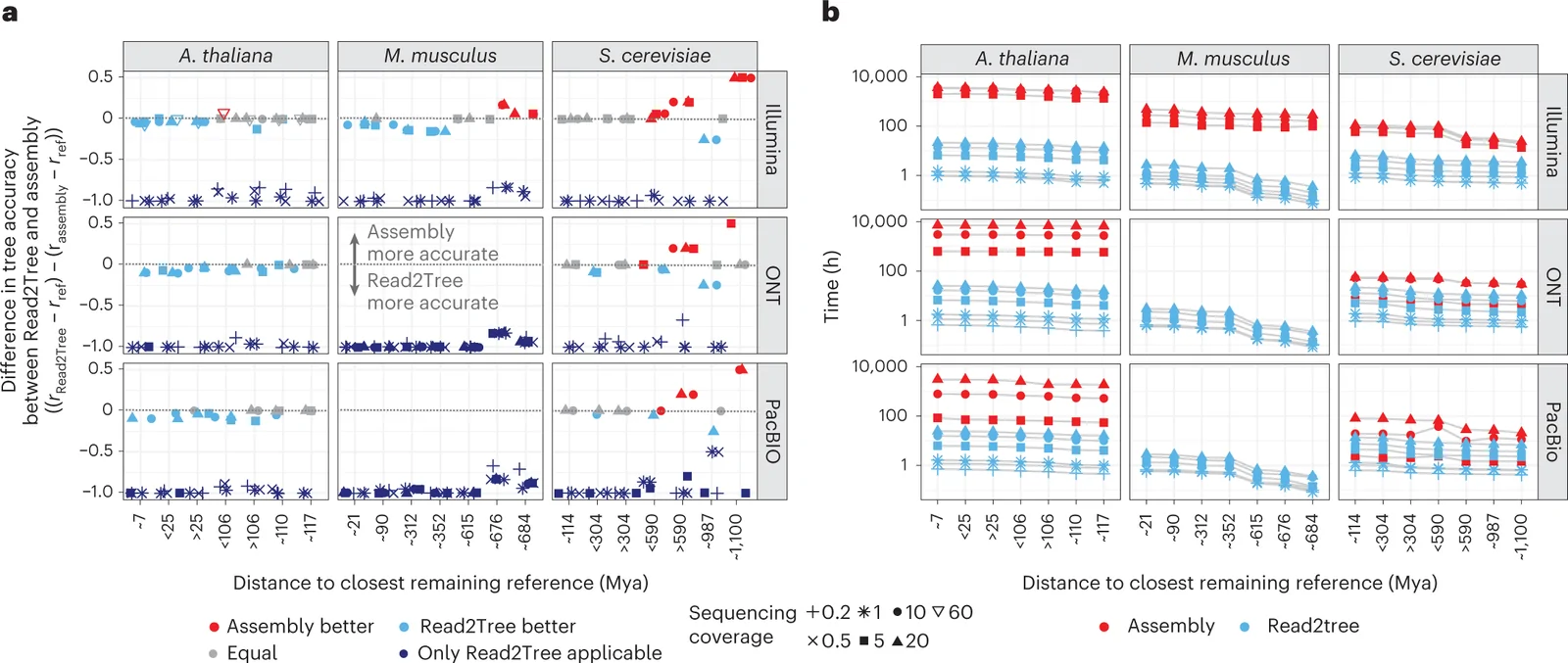
A comparison of Read2Tree with a regular pipeline with assembly, orthology prediction and MSA computation. **a**, A comparison of trees using the difference between the reference tree and either the tree of Read2Tree or the tree coming from the assembly approach. For dark blue, we had only Read2Tree trees as assemblies for these low coverages are not possible to obtain. Below zero (in dark or light blue), Read2Tree is more accurate, while above zero (in red), the assembly approach is more accurate and gray indicates no difference between the methodologies. **b**, A comparison of wall time needed from reads to availability of concatenated MSA showing the dependencies of available closest remaining reference and coverage.

**Fig. 4:**
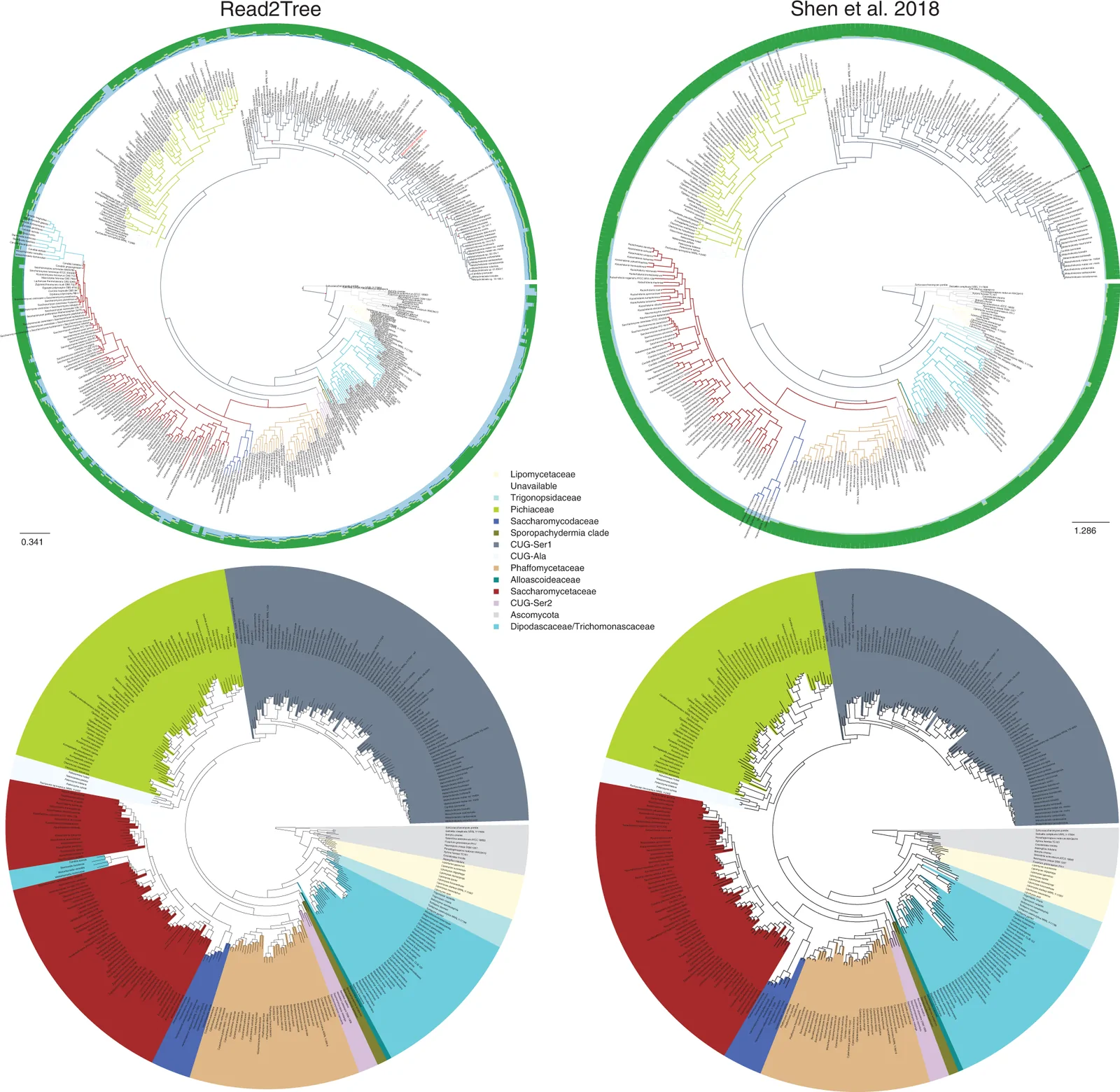
High consistency between Read2Tree and a state-of-the-art phylogenetic pipeline^31^. The top row shows the full trees and the alignment matrix used to compute the tree as outer circles. The red dots indicate nodes with a bootstrap below 100. The species *Naumovozyma dairenensis*, previously misclassified^32,33^, is highlighted in red. The bottom row shows trees trimmed to an overlapping leaf set.

**Fig. 5:**
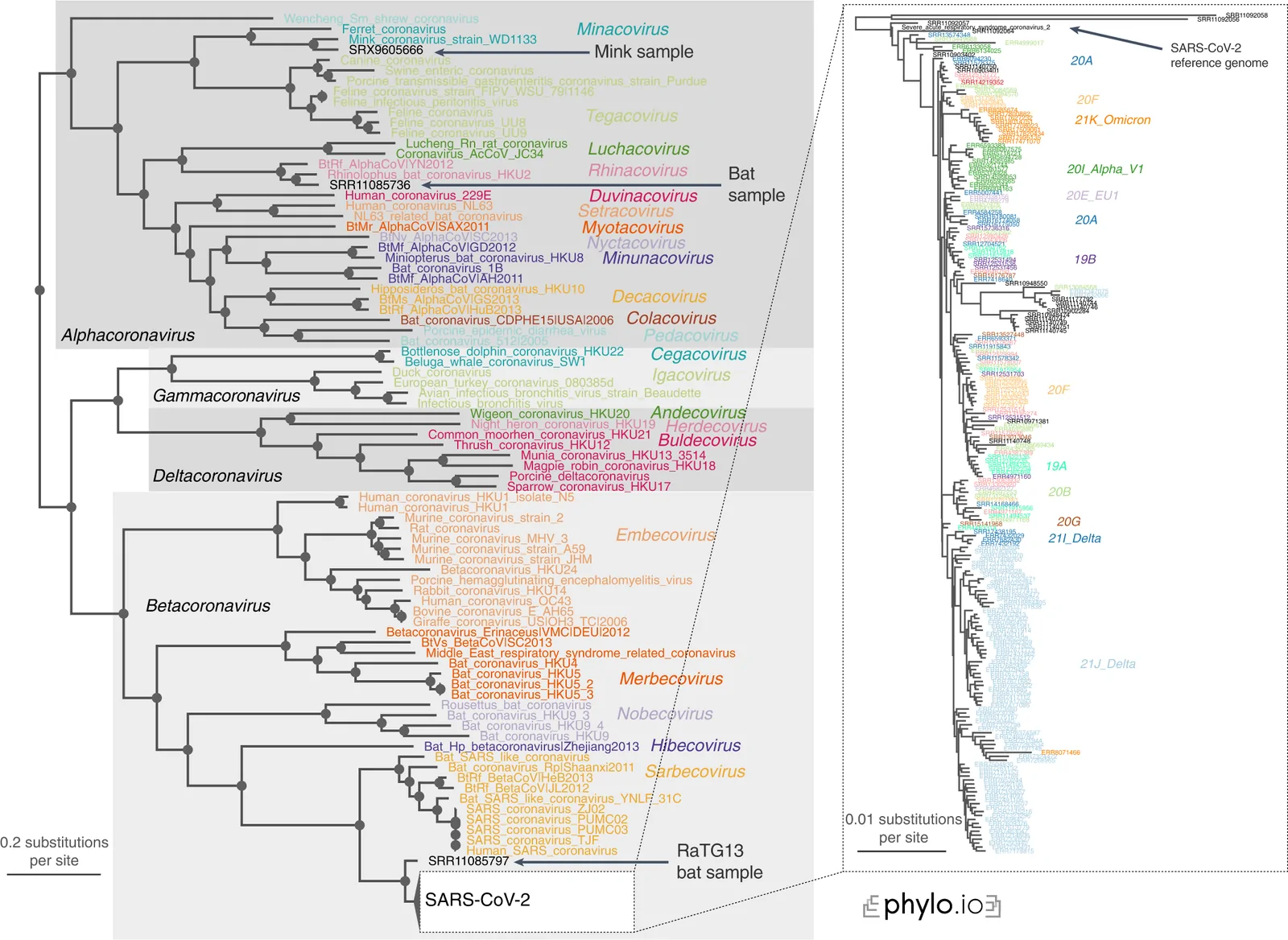
Read2Tree correctly classifies the recent SARS-CoV-2 sequences and recapitulates the evolution of the individual variants. All genera (gray boxes in the overall tree) and subgenera (colored labels) are correctly delineated. The inset focuses on the part of the tree with 215 SARS-CoV-2 samples, and variants of concern (colored labels) cluster consistently on the tree, indicating that Read2Tree can be used to categorize the samples.

State-of-the-art phylogenomic pipelines require many steps, which can be both time consuming and error prone (Fig. 1a). With Read2Tree, we directly process raw sequencing reads and reconstruct sequence alignments for conventional tree inference methods (Fig. 1b and Supplementary Fig. 1). We start by aligning raw reads to nucleotide sequences derived from the genome-wide reference orthologous groups (OGs; we used Mafft^20^ as default) (Fig. 1b, 1). Within each OG, we reconstruct protein sequences from reads aligned to reference sequences (Fig. 1b, 2). Importantly, these sequences in reference OGs are not restricted to single-copy marker genes, such as the mitochondrial cytochrome *c* oxidase I gene or BUSCO genes^21^; they also include multiple paralogous genes as well as nonuniversal genes. This is achieved by leveraging OGs computed from 2,500 diverse genomes analyzed in the Orthologous Matrix (OMA) resource developed in our laboratory^22,23^. Next, we retain the best reference-guided reconstructed sequence, using the number of reconstructed nucleotide bases as criterion (Fig. 1b, 3 and Supplementary Fig. 2). Subsequently, the selected consensus is added to the OG’s multiple sequence alignment (MSA) (Fig. 1b, 4). Finally, putative OG selection and tree inference can proceed using conventional methods (we use IQTREE^24^ by default; Fig. 1b, 5). For greater detail on the individual steps, see Methods.

This way, Read2Tree is able to report key information across putative OGs in a fraction of the time over conventional comparative genomic pipelines—by bypassing genome assembly, annotation, homology and orthology inference. Furthermore, because each sample is processed independently, Read2Tree can process the input genomes in parallel, and scales linearly with respect to the number of input genomes.

### Impact of coverage and distance to reference on accuracy

We tested Read2Tree on a wide array of conditions, with two kinds of sequence (DNA versus RNA), three target species (*Arabidopsis thaliana*, *Saccharomyces cerevisiae* and *Mus musculus)*, three types of sequencing technology (Illumina, PacBio and Oxford Nanopore Technologies (ONT)), six levels of sequencing coverage (ranging from 0.2× to 20×) and six different sets of reference species (increasingly distant from the targets spanning over 1 billion years of evolution) (Fig. 2a). For sequence reconstruction accuracy (Fig. 2b), we measured both the correctness of the reconstructed sequences (‘precision’) and the completeness of the reconstructed sequences (‘recall’). For tree reconstruction accuracy (Fig. 2c and Supplementary Fig. 6), we compare the reconstructed tree with the known species phylogeny and report both the precision and the recall of the reconstructed trees, in terms of the branches with at least 90% support.

In general, Read2Tree was able to maintain a high precision in terms of sequence reconstruction (Fig. 2b) and tree reconstruction (Fig. 2c) across all datasets, with varying levels of recall depending on the dataset difficulty. First, we assessed the effect of coverage ranging from 0.2× to 20× of the individual datasets. We observed that increasing the sequencing coverage had little impact on precision, and mainly lowered recall: in most configurations, Read2Tree could maintain 90–95% precision at the sequence level even with coverages as low as 0.2× (Fig. 2b). The best low-coverage results were obtained on transcriptomic short-read data in mice, where precision reached 98.5% at 0.2× coverage. To assess the versatility of Read2Tree, we benchmarked it across DNA and RNA datasets. This did not have a large impact in general, but transcriptomic RNA results (in the mouse dataset) are marginally less impacted by differences in average coverage, perhaps due to the large coverage variance from uneven gene expression levels in these data (Fig. 2b,c). Next, we assessed whether Read2Tree is capable of utilizing the range of current sequencing technologies. For this, we applied it across traditional short reads, Oxford Nanopore and PacBio long reads. To enable this, Read2Tree has slightly different mapping strategies built in for long versus short reads (Methods). As Fig. 2b,c shows, Read2Tree maintained a high accuracy across each sequencing technology, but we observed the highest accuracy over traditional short reads. We have not assessed more recent sequencing technologies such as PacBio HiFi or Illumina infinity that might change this result.

Finally, we assessed the robustness of Read2Tree with respect to the evolutionary distance between the sample at hand and the closest relative in the reference set. This is often critical as one might not know the closest ancestor that is assembled or it is not available^25^. Thus, we tested Read2Tree across a wide range of evolutionary distances ranging from 7 million years ago to over 1.1 billion years ago. While these are certainly extreme scenarios, overall Read2Tree was able to cope with them successfully. Figure 2b,c shows that the choice of reference set mainly impacted recall, with closer reference genomes leading to more reconstructed positions. Remarkably, Read2Tree was able to maintain high accuracy even in the datasets with very distant references—for example, processing mouse RNA sequencing (RNA-seq) data without any vertebrate genome in the reference set.

We also tested Read2Tree on simulated data, for coverages between 0.1× and 10× and distance to the closest reference varying between 2 and 150 point accepted mutation (PAM) units—where 100 PAM corresponds to one substitution per site on average. The reconstructed trees were perfect in all but the most extreme scenarios (PAM >120 or coverage <0.5×; Supplementary Fig. 7).

Given the extensive benchmarks across species, coverage, sequencing technology, assay (DNA and RNA) and simulated data, we observe that Read2Tree is indeed a highly versatile and accurate tool to reconstruct phylogeny directly from raw reads.

### Faster and often more accurate than assembly-based trees

Next, we compared the performances of Read2Tree with conventional assembly pipelines. For this, we generated de novo assemblies and protein predictions across the same datasets as from the previous section, using Canu^26^ for PacBio and ONT data and Megahit^27^ together with SoapDeNovo^28^ for the Illumina reads (Methods). The conventional assemblies were processed using OMA standalone, including the same exported reference genomes, as OMA standalone was previously shown to identify the most accurate phylogenetic marker genes^29^. For the inclusion of orthologous markers in the concatenated alignment used for tree inference, we required a commonly set minimum threshold of 80% taxon presence. As above, we varied the closest remaining species in the dataset by removing species along the reference tree (Fig. 2a). With different coverages and reference sets, we obtained 42 data points per species. For each of these data points, we performed the orthology inference separately and recorded its computation time. The proportion of sequences placed into the respective OGs showed high levels of variation (Supplementary Fig. 8a). For each assembly and variation of proteomes, we computed the topological distance between the resulting tree from assembly or Read2Tree with trees obtained using high-quality genome assemblies for *A. thaliana* and *S. cerevisiae*.

Figure 3 shows the overall results, highlighting the performance of Read2Tree. Perhaps unsurprisingly, we observed that coverage levels had a profound impact on the performance of assembly-based approaches, rendering them incapable of dealing with coverages below 5–10×. Thus, for these datasets, we report only Read2Tree results.

Where both approaches can be compared, the only cases where the conventional de novo assembly approach outperformed Read2Tree were with high coverage and very distant (>500 Mya) to the closest reference species (Fig. 3a, upper right region of each graph). In all other scenarios, Read2Tree outperformed the conventional approach in accuracy. Specifically, on the yeast dataset at a higher coverage level, both assembly and Read2Tree performed well overall—we never observed more than two different branches between the obtained and reference trees. With at least 10× coverage and distant reference species, the conventional assembly approach outperformed Read2Tree (Fig. 3a and Supplementary Fig. 4).

By contrast, on the more complex *A. thaliana* and *M. musculus* datasets, Read2Tree outperformed the assembly approach—with fewer differences to the reference (up to two different branches for Read2Tree, versus up to four for the conventional approach). On the ONT data—characterized by longer reads but higher error rate—Read2Tree outperformed the conventional approach on both datasets.

Finally, in terms of compute time, Read2Tree was generally much faster than the conventional approach, up to 100 times faster on the larger genomes (Fig. 3b and Supplementary Fig. 8b).

Altogether, these results indicate that Read2Tree is faster in all conditions, and produces reliable trees in low-coverage datasets and other datasets where the conventional approach fails entirely (long-read transcriptomics). At higher coverage levels, the trees inferred by Read2Tree rival in quality those obtained from assembled reference species with a full pipeline, particularly when applied to more complex genomes, and unless the closest reference species is very distant (>500 million years).

We also compared Read2Tree with Mash, a fast *k*-mer-based approach^30^ commonly used on bacterial genomes. While the alignment-free approach of Mash was much faster than even Read2Tree, the resulting trees were much less accurate than either Read2Tree or the assembly-based approach (Supplementary Fig. 5). This illustrates why alignment-free approaches such as Mash, while very useful for fast approximations, are typically not suitable to reconstruct high-quality phylogenetic trees.

### Accurate reconstruction of a 435 species yeast tree of life

To assess a potential large-scale application for Read2Tree, we applied it to reconstruct a large yeast phylogeny from raw reads. Thanks to Read2Tree’s ability to process low-coverage datasets, we could extend our analysis to all Illumina single- and paired-end, ONT, PacBio and 454 sequencing read datasets available for budding yeast in the NCBI Sequence Read Archive (SRA) database (November 2018, 404 species) and 31 reference species obtained from the OMA database (release 2018, 3,063 OGs). Using an automated approach for retrieval and mapping, we were able to obtain direct sequences for 404 species (Supplementary File 1). Read2Tree could process these datasets in around a month of computation (adding each species sequentially and performing the mapping on 30 central processing units (CPUs)—one CPU per reference—in parallel), due to its ‘embarrassingly parallel’ architecture, with every sample being processed independently up to phylogenetic inference (10× Illumina: ~20 min using four threads).

A large proportion of these datasets were recently used to construct a phylogeny across 363 budding yeast species^31^. This included a dataset of 196 new assemblies and their annotations^31^. This large effort provided a delineation of the yeast tree of life into 13 main clades and highlighted the influence of horizontal gene transfer in the evolution of yeast species^31^. Due to the complexity of state-of-the-art pipelines, it also consumed millions of CPU hours and years of work. Furthermore, the conventional assembly-based approach could not include low-coverage samples into their analysis. We were able to extend this work using Read2Tree using a fraction of the resources.

Using Read2Tree, we were able to compute and produce this large phylogeny across 435 samples (including 31 species as reference). Some of the samples failed due to their too low coverage levels of around 3.1× assuming a 12-Mbp-long average genome size. Nevertheless, using Read2Tree we were able to include multiple samples even at coverage levels below 5×, which were reported with over 2,500 sequences placed in OGs (Supplementary Fig. 14). Read2Tree was able to reconstruct the phylogeny and also reported the phylogeny-relevant genes assembled per sample, which overall showed similar GC levels as the reference data (Supplementary Fig. 15). This was also exemplified by the fact that we did not observe a correlation between the number of sequences placed into OGs per species and their individual coverage (Supplementary Fig. 14, correlation 0.2).

Considering the subset of species in common, our results were highly congruent with those of Shen et al.^31^ (Fig. 4 and Supplementary Figs. 12 and 13): both trees exhibited similar distances to the NCBI taxonomy tree—297 splits in ours versus 291 splits in Shen et al. In direct comparison, Shen et al. and Read2Tree were more similar with one another, with only 128 different splits (20% difference of the branches), than either was to the NCBI taxonomy. After collapsing branches with a support below 90, the difference in the number of splits between the conservative NCBI tree and ours was 29 splits, and 25 splits between ours and Shen et al. Twenty-four of these splits were in common between Read2Tree and Shen et al. To get more insight into the nature of these differences, we assessed the agreement with the NCBI taxonomy for two different levels of resolution: family and genus. At the coarser family level, Read2Tree was more consistent with the NCBI taxonomy for six families, while Shen et al. was more consistent in one family (Supplementary Fig. 10). At the finer genus level, Read2Tree was more consistent with the NCBI taxonomy for four genera, versus ten for Shen et al. (Supplementary Fig. 11).

Nevertheless, there are still certain differences between Read2Tree and the NCBI taxonomy remaining. While resolving most such instances would constitute entire follow-up studies in their own right, we were able to explain one apparent disagreement: *Naumovozyma dairenensis* is placed in the CUG-Ser1 classification, while according to the NCBI taxonomy, it should be an ascomycetous yeast in the *Saccharomyces* sensu lato group within the family Saccharomycetaceae. However, this is a case of erroneous metadata reported in the literature^32,33^.

Given this phylogeny, we can now easily update and extend it using Read2Tree in a matter of minutes with additional sequences being generated. This enables a deep dive into the comparative genomics of yeast and to further explore their differences between the strains and their impact on life, food production and so on. This is also easily reproducible for other organisms as Read2Tree is capable of spanning large evolutionary distances with respect to the reference tree.

### Read2Tree for zoonotic surveillance and human epidemiology

To further illustrate the versatility of Read2Tree, we used it to reconstruct a phylogeny encompassing various coronaviruses from the OMA coronavirus database, as well as 215 raw coronavirus sequencing samples deposited to the SRA. Besides the putative SARS-CoV-2 sequence, we also included two samples from bat (SRR11085797 (ref. ^34^) and SRR11085736 (ref. ^35^)) and one from mink^36^ (SRX9605666).

The reconstructed phylogeny was in complete agreement with the lineage classification obtained from the UniProt reference proteomes. In particular, the tree recovered not only the main coronavirus genera (*Alpha*-, *Beta*-, *Gamma*- and *Deltacoronavirus*) but also all subgenera with complete consistency (Fig. 5).

The first bat sample corresponds to the reads of RaTG13, which is the closest relative of SARS-CoV-2 identified yet^34^. Indeed, in our tree it falls right outside the SARS-CoV-2 clade. The other bat sample could also be confirmed as an *Alphacoronavirus*, subgenus *Rhinacovirus*^35^. Likewise, we could confirm the classification of the mink sample, identified as an *Alphacoronavirus*, subgenus *Minacovirus* by the authors^36^.

The position of the SARS-CoV-2 sequences within the coronavirus tree of life is also consistent with our prior knowledge on them. The reference genome, the Wuhan-Hu-1 sequence reported in early January 2020 (ref. ^37^), is at the base of the subtree. The only three sequences that branch out before it are SRR11092056-8—which were obtained from patients with severe pneumonia at the beginning of the pandemic^34^. Finally, we note that the variants of concern included in the analyses appear clearly as distinct clades on the tree.

To empirically test the scalability of our method, we also used Read2Tree to process 10,283 SARS-CoV-2 samples. The reconstructed tree clustered the sequences according to Centers for Disease Control variants of concerns classification, providing further evidence that the tool can be used to quickly and reliably classify SARS-CoV-2 variants (Supplementary Fig. 17). The same observation held for additional controls—running Read2Tree using coding-gene markers only (Supplementary Fig. 16), and using FastTree^38^ as the tree inference method (Supplementary Fig. 18).

Overall, this application of Read2Tree to diverse coronaviruses sequences illustrates the ability of the tool to deal both with the considerable phylogenetic breath of this family of virus^39^ and the depth required to classify individual SARS-CoV-2 variants of concerns. This makes Read2Tree suitable for both zoonotic surveillance and human epidemiology^40^.

## Discussion

We presented Read2Tree, an approach to scale and ease the laborious process of comparative genomics: assembly, annotation and phylogenetic comparison. These steps are computationally costly and error prone and require specialized knowledge. Using Read2Tree, we can directly reconstruct phylogenetic-relevant genes from raw reads, and thus enable a placement and comparison of the species at hand with minimum computing and coverage requirements. The efficiency of the approach makes it possible to process a large number of samples in parallel, using a consistent methodology and without compromising accuracy compared with state-of-the-art pipelines.

Current inherent problems of large-scale comparative genomics, or in general comparative genomics projects, recently shifted from obtaining accurate assemblies to annotation and curation of these assemblies. This was in part possible due to sequencing technology advancements over long reads^16,18^, but also due to innovations in assembly algorithms^41,42^. These steps still require high DNA quality and are in general more expensive, but enable large projects such as the Vertebrate Genome Project^43^, the human pangenome^44^ and telomere-to-telomere^45^ projects. Nevertheless, in all of these cases, the annotation of the genomes and the improvements in terms of continuity and accuracy remain major bottlenecks. Additionally, we showed that Read2Tree enables accurate analysis across all three sequencing technologies (Illumina, ONT and PacBio), in a fraction of the time. Furthermore, large-scale consortia could also benefit from running Read2Tree, despite having high-coverage datasets, to independently quality control (QC) their assembly and tree building approaches.

One major advantage is that, despite side stepping de novo assembly, Read2Tree can operate in the absence of close reference genomes; indeed, we demonstrated accurate tree reconstruction involving sequencing reads from species separated by hundreds of millions of years of divergence. Though we also reached some limits to this robustness, when subjecting Read2Tree to both very high divergence and low sequencing coverage, it should be noted that evolutionary distances will tend to diminish as ever more species get sequenced across the tree of life.

Furthermore, while most authors of genome resources deposit annotation sets alongside the assembled sequences, not all of them do. The ability to process genomes directly from raw reads not only circumvents this limitation, but it can also reduce the biases arising from overreliance on specific reference genomes. There have been some initial efforts to ‘dehumanize’ nonhuman great ape genomes^46^, but many other clades still suffer from analogous biases, which can be greatly reduced by processing raw reads.

We demonstrated the speed and accuracy of Read2Tree over a large-scale yeast dataset. Here Read2Tree was able to reconstruct a high-quality tree from raw read samples directly retrieved from the SRA. This was achieved despite variation in coverage levels and other possible technical biases.

In a second illustrative application, we reconstructed a tree from raw coronavirus sequencing data, including 10,000 samples from the ongoing SARS-CoV-2 pandemic. Here Read2Tree was again able to classify and place all samples correctly, be it across the full breadth of the *Coronaviridae* family, or across the depth of minute variations among SARS-CoV-2 samples, where the optimal choice of phylogenetic marker genes typically depends on the level of sequence divergence^47^.

We also compared Read2Tree with an ultrafast, alignment-free approach (Mash) where Read2Tree achieved a much higher accuracy (Supplementary Fig. 5). In its current form, Read2Tree serves a distinct function from metagenomic classifiers such as Kraken2 (ref. ^48^) or Centrifuge^49^. Indeed, while these tools seek to exploit known characteristic sequences for read-level taxonomic classification, Read2Tree aims at efficiently extracting the genome-wide (or transcriptome-wide) phylogenetic signal by inferring large multi-locus input data matrices for phylogenetic tree inference tools, a step that has been shown to be critical for resolving difficult phylogenies^17,29,50–52^. Nevertheless, Read2Tree could be further developed to process metagenomic samples—by combining it with a genome binning preprocessing step. In recent years, a number of different approaches for genome binning have been proposed, be it through ‘differential coverage’ approaches, which exploit correlated abundance across samples to identify reads coming from the same species^53–55^, using Hi-C protocols, which make it possible to identify parts of DNA in close physical proximity^56,57^, or single-cell technologies^58^.

Overall, Read2Tree is an approach for reconstructing phylogenetic important genes and characterizing the sample at hand or entire sample collections, enabling the study of a large number of genes and their evolution with no preprocessing, few computational resources and minimal bioinformatic expertise. This will enable faster and more comprehensive phylogenetic reconstruction efforts—from tiny virus genomes to large eukaryotic ones, but also cell lineage, cancer trees and other kinds of phylogenies across biology and medicine.

## Methods

### Description of the Read2Tree method

Read2Tree incorporates various publicly available tools for some of its steps (MAFFT^20^, NextGenMap^60^ and Samtools^61^) and uses these in a structured manner to go from reads and reference OGs to a concatenated alignment that is fed directly into a tree inference tool, which by default is IQTREE^24^. For this purpose, it needs two sets of input data: (1) a set of reference OGs that can be obtained directly from the OMA database and (2) the reads to be mapped coming from a single species. The Read2Tree pipeline works in the following way. First, it retrieves DNA sequences using the REST-API from the OMA browser from the selected reference OGs, then sorts these into one file per species. In parallel, it computes alignments using the AA sequence with MAFFT^20^, and then uses the codon information to generate DNA alignments. Once computed, all reads are mapped against the DNA reference species and a consensus sequence is constructed (local assembly). Since our local assemblies are reference guided, they can never be longer or shorter than the longest or shortest sequence part of an OG. Local assemblies are then placed into the alignments using the coordinates of the best selected reference. Therefore, no new alignment is necessary and we can assure that the right AA/DNA is placed in the right position in the alignment. The resulting alignments for each OG are then concatenated and a tree is computed. More details about the inner workings of Read2Tree are provided in Supplementary Fig. 1.

Read2Tree can be parallelized using multiple instances across the mapping step. It is recommended to compute the reference set first. The mapping step can then be split such that each mapping can be performed as single job submission on high-performance clusters.

### OG selection

OGs were selected from OMA^62^ using the marker gene export functionality (https://omabrowser.org/oma/export_markers/). For all species, the maximum number of covered species was set to 0.8 and the maximum number of markers to −1 (unlimited). Species selected are displayed in Fig. 1a.

### Reads

Whole genome sequencing reads for *A. thaliana* and *S. cerevisiae* were obtained from the SRA database for technologies PacBio, Illumina and Oxford Nanopore. Messenger RNA-seq reads for *M. musculus* were also obtained for all three technologies from the SRA database. Subsampling of reads was performed in Python (ref. ^63^). For PacBio and ONT reads, subsampling was optimized such that the cumulative number of bases fits to the expected coverage. For the coverage test, reads were subsampled assuming a 38 Mbp accumulated gene length (transcriptome) for mouse, and 120 Mbp thale cress and 12 Mbp yeast genome lengths. Reads were sampled to obtain 20×, 10×, 5×, 1×, 0.5× and 0.2× coverage levels. Reads for the big yeast tree were obtained from the SRA database (Supplementary File 1). Reads for coronavirus were obtained from the SRA database (Supplementary File 1). All SRA numbers are available in Supplementary File 1.

### Reference tree construction

Reference trees for the three evaluated species were computed using the species as defined in Fig. 2a. Species were selected from OMA^62^ as described in the OG selection. Individual OGs (gene markers) were aligned using MAFFT^20^ version 7.310 (–maxiter 1000–local), and trees were inferred with IQTREE^24^ version 1.6.9 (-m LG -nt 4 -mem 4 G -seed 12345 -bb 1000). For reference trees that were used for testing the dependency on the reference dataset, specific species were deleted from existing alignments and trees were computed with IQTREE as stated before. All reference trees are available in Supplementary File 2. To highlight the years of evolution, we collected the time using timetree^59^ (April 2022).

### Read2Tree runs

For the single species runs (Figs. 2 and 3), Read2Tree was run with default parameters. For the large yeast tree (Fig. 4), Read2Tree was run in multiple steps. First, the reference dataset was obtained (using the –reference option). Then, mapping was parallelized such that for each species the mapping against a single reference was performed individually (using –single_mapping option). This means that, for each species, 31 parallelized mappings were performed. Additionally, species with reads with more than 20× coverage were sampled to 20× coverage assuming a genome length of 12 Mbp. Subsampling of reads is integrated into the Read2Tree workflow. Finally, all mapped species were merged together and concatenated to provide the multiple sequence output (using –merge_all_mappings).

### Accuracy assessment

We assessed the accuracy of sequence reconstruction by taking each Read2Tree reconstructed sequence (for each species, coverage, technology and removal level) placed in an OG and performed a blastp (ncbi-blast, version 2.8.1) search against its original OG that contained the original sequence coming from a high-quality assembly for the species of interest. The accuracy was measured as the blast percentage identity and recall as the total number of obtained amino acids in the concatenated MSA of all OGs. Additionally, we evaluated whether the top hit of the Read2Tree reconstructed sequence was most similar to its assembled same-species counterpart, or the sequence used as reference for reconstruction or any other random sequence part of that particular OG (Supplementary Fig. 3).

### Assemblies

For the three species, whole-genome data were assembled with individual sequencing technology specific assembly programs, following best practice or default parameters. For Illumina, we first used megahit^27^ (version 1.2.9) with default parameters for assembling the contigs. Subsequently, SOAPdenovo^28^ (version 2.04-r241) was used for scaffolding: first, SOAPdenovo-fusion -D -K 41 -c megahit.contigs.fa -g scaffold_prefix -p 20 followed by SOAPdenovo-63mer map and scaff with recommended parameters over the config file. For ONT reads, we assembled the reads using Canu^26^ (version 2.0) with a specified genome size (genomeSize) gnuplotTested = true -nanopore-raw and useGrid = false parameters to run it locally on only one node on the cluster. Lastly, for PacBio continuous long reads data, we also used Canu (version 2.0) with similar parameters, but specifying the -pacbio-raw parameter. All run times were measured using linux time, and the wall and CPU time were recorded. The RNA-seq data were assembled differently to the whole genome. For Illumina RNA-seq, we used Trinity^64^ (version 2.8.5) with the following parameters: –seqType fq–max_memory 50 G–left reads1.fq.gz–right reads2.fq.gz–CPU 6–trimmomatic–full_cleanup–output prefix. These execute Trimmomatic automatically and follow the recommendations from Trinity.

### Orthology prediction of assembled genomes

For each assembly (species, technology and coverage level), we ran OMA standalone (version 2.3.3) on the UNIL HPC clusters using a Slurm scheduler. For this, we collected all the species as depicted in Fig. 2 using the OMA All versus All export function. Then we removed the relevant species according to Fig. 2, adding each time the assembly for mouse, yeast or thale cress in the set and running the orthology prediction with standard parameters (OMA version 2.2.1). Thus, for instance, for the Illumina *M. musculus* 10× assembly, we ran OMA seven times for all reference datasets with increasing distance to its closest relative. In total, we ran 126 different OMA runs with seven variations of reference proteomes and three variations of technologies, with three coverage levels for *A. thaliana* and *S. cervisiea*. Additionally, we ran OMA 21 times for *M. musculus* 5×, 10× and 20× Illumina assemblies. The all-versus-all part was parallelized on 1,000 nodes, and the final part was run on a single node with 40 G memory. To obtain OGs for tree inference, we applied the 0.8 taxonomic occupancy threshold, as previously. OGs were filtered according to the procedure in Shen et al. (see below). OGs were individually aligned using MAFFT^20^ version 7.310 (–maxiter 1000–local) and concatenated, and trees were inferred with IQTREE^24^ version 1.6.9 (-m LG -nt 4 -mem 4G -seed 12345 -bb 1000).

### Tree-versus-tree comparison

Each Read2Tree tree was compared with a fitting reference using several tree distance measures. For topological similarity, we used two approaches, one that uses the Robinson–Foulds distance and counts the number of different splits between two trees and one that collapses each node with a bootstrap support below a certain threshold and then counts the number of overlapping splits. Then, we define as recall the number of overlapping splits divided by the number of splits in the reference and precision as the number of overlapping splits divided by the number of splits in the Read2Tree tree.

### Large yeast tree

For the large yeast tree, we extracted all available yeast datasets from the SRA in November 2018 (406 species, Supplementary File 1) and applied Read2Tree (standard parameters) to 31 yeast species extracted from the OMA database (November 2018) using the marker export function with minimum species coverage of 0.8 (3,082 OGs). The selected species are available in Supplementary File 3. Reads from the SRA database were mapped according to their sequencing methodology using Read2Tree. To compare our analysis with Shen et al., we aimed to have as many species in common as possible. For this purpose, we complemented our tree with sequencing reads that we simulated from assembled genomes for 15 species that were present in the tree of Shen et al. but were missing from our dataset (Supplementary File 1). Simulations were conducted with InSilicoSeq (version 1.3.0 https://github.com/HadrienG/InSilicoSeq, –model hiseq -n 600000). To map the species from the tree of Shen et al.^31^ to our tree, we obtained the taxon identification of species/strains using NCBI interface of ete3 (ref. ^65^). For species where automated mapping was not possible, we obtained the taxon identification using the NCBI taxonomy interface (https://www.ncbi.nlm.nih.gov/Taxonomy/Browser/wwwtax.cgi).

### Filtering OGs yeast as in Shen et al.

Given the reconstructed sequences placed in their respective OGs and added to their alignment, we decided to compute a tree following the protocol of ref. ^31^. In brief, from the 3,082 alignments, we selected those that contained more than 171 species, resulting in 1,829 OGs. Then, we used phyutils 2.2.6 (seqs -aa -clean 0.01) to clean up the alignments. Since our approach does not place multiple sequences from the same species into one OG, we skipped the removal of putative paralogs. Within the alignments we changed all ‘X’ with the gap character ‘-’. Then, we applied trimAl version 1.4.rev15 (-gappyout). Next, we removed protein sequences with lengths shorter than 50% the length of the trimmed MSA length of each OG they belonged to. We also removed OGs in which the total trimmed MSA length was <167 amino acid sites. These resulted in 926 alignments. With these alignments, we used IQTREE (version 1.6.9) with automatic model selection to compute trees. Then, we identified species in the gene trees that had a branch length longer than 20 times the median of all branch lengths. We removed these species from the respective alignments, again controlling that more than 171 species are included. We then computed the tree using IQTREE (-seed 12345, -m LG + G4, -bb 1000, -nt 20).

### Large yeast tree comparison

Using all taxon identifications, we retrieved the current NCBI reference taxonomy and the classification of each species. We then compared the three trees (NCBI, Read2Tree and Shen et al.^31^) using the Robinson–Foulds distance on the overlapping leaf set. Additionally, we overlaid the Shen et al. classification on our tree. Finally, we compared the trees using the ancestral node that contains the highest number of monophyletic species given a specific grouping (order, family and phylum) extracted from the NCBI taxonomy information. All comparisons were conducted using custom Python Jupyter notebooks. Additionally, we collected data on GC content and the input coverage-to-mapping ratio. Trees were visualized with ete3 (ref. ^65^). The tanglegram plot was produced using the dendextend R library^66^. A side-by-side topological comparison was obtained using phylo.io^67^.

### *Coronaviridae* tree reconstruction

Marker genes were exported from https://corona.omabrowser.org/ with at least four species. DNA sequences for these genes were obtained from the same resource. Four extra groups with intergenic regions from the SARS-CoV-2 reference genome were added using a custom script. We extracted consecutive chunks of at least 30 bp from the reference genome MN908947 assembly that were not covered either by any CDS region or proteins not belonging to any OMA group in the https://corona.omabrowser.org resource (that is, ORF8 and ORF10). This led to four regions (1..265; 26473..26522; 27760..27893; 29675..29903) that we treated as additional groups. SARS-CoV-2 samples were obtained from Nextstrain open (https://data.nextstrain.org/files/ncov/open/global/metadata.tsv.xz)^7^. Different samples with SRA accessions that span all different clades were obtained with a custom Python script (included in the linked repository below). SRA read accessions together with the clade annotations from Nextstrain are available in Supplementary File 1. Reads were downloaded from the SRA database and trimmed. Read2Tree was applied to this dataset, and all obtained reads were mapped to the marker genes. Read2Tree was run with standard parameters. The resulting supermatrix alignment was filtered by removing columns that had more than 70% gaps. This removed 30,969 columns resulting in a supermatrix of size 295 × 42,669. Finally, the tree was inferred using IQTree2 (ref. ^24^) (version 2.2.0-beta) with parameters -m GTR -ninit 2 -me 0.05. As additional controls, we computed the trees with FastTree^38^ version 2.1.11 instead of IQTREE2 and without the additional four extra groups. All trees are available in Supplementary File 1.

For the scaled-up experiment with 10,283 samples, we used the same protocol, except for the source of the read annotations. Here we used the clade annotations from https://harvestvariants.info/ (accessions and annotations are available in Supplementary File 1).

### Simulated phylogeny analysis

The simulated phylogeny includes a fixed topology for species tree with 15 species using the ALF package^68^ (version 0.99). We varied the branch length leading to one of the species (species of interest) to between 2 PAM and 150 PAM. For each run, we infer afterwards the OMA groups (excluding the species of interest). Then, using art_illumina^69^, we generated DNA sequencing reads (paired end) with length of 100 and 150 bp and coverage of 0.1 to 10. Next, for each case, we ran Read2Tree to infer the phylogeny. Finally, we calculated the Robinson–Foulds metric between inferred species tree and the true one on the basis of the output of ALF.

### Comparison with Mash

We took established assemblies as a reference that we downloaded from NCBI. Subsequently, we used Mash (version 2.3) sketch^30^ with a size of 10 m (*k* = 21 as default), followed by Mash distance to obtain distances between the genomes, and analyzed the reads against that reference set. Finally, we applied RapidNJ^70^ (version 2.3.2) on the distance matrix obtained from Mash to infer the species tree. We did that for different distances across the references that were provided, always comparing the reads from, for example, *A. thaliana* with the assemblies.

### Reporting summary

Further information on research design is available in the Nature Portfolio Reporting Summary linked to this article.

## Online content

Any methods, additional references, Nature Portfolio reporting summaries, source data, extended data, supplementary information, acknowledgements, peer review information; details of author contributions and competing interests; and statements of data and code availability are available at https://doi.org/10.1038/s41587-023-01753-4.

## Supplementary information


Supplementary InformationSupplementary Figs. 1–18.
Reporting Summary
Supplementary Data 1Accession numbers for all analyses (SARS-CoV-2 standard and 10,283-samples dataset, yeast tree and accuracy benchmark).
Supplementary Data 2Reconstructed phylogenetic trees for the accuracy benchmark and SARS-CoV-2 analysis (Newick string format).
Supplementary Data 3Details on the reference genomes used for the accuracy benchmark.


## Data Availability

References used and all SRA numbers of reads used are available in Supplementary File 1. Supplement, scripts and reference data are available at https://github.com/dvdylus/read2tree_paper (ref. ^59^).

## Code availability

The source code for Read2Tree is available under an MIT open-source license at https://github.com/DessimozLab/read2tree (ref. ^71^).
