# Supplementary material for: Inference of phylogenetic trees directly from raw sequencing reads using Read2Tree
Source: Nat Biotechnol. 2023 Apr 20;42(1):139–47. doi: 10.1038/s41587-023-01753-4 (PMC10791578; doi:10.1038/s41587-023-01753-4)
Supplement: Supplementary file 1 — Supplementary Figs. 1–18. [file 41587_2023_1753_MOESM1_ESM.pdf]

nature biotechnology

Article https://doi.org/10.1038/s41587-023-01753-4

Inference of phylogenetic trees directly from raw sequencing reads using Read2Tree

In the format provided by the authors and unedited

Read2Tree — Supplementary Figures

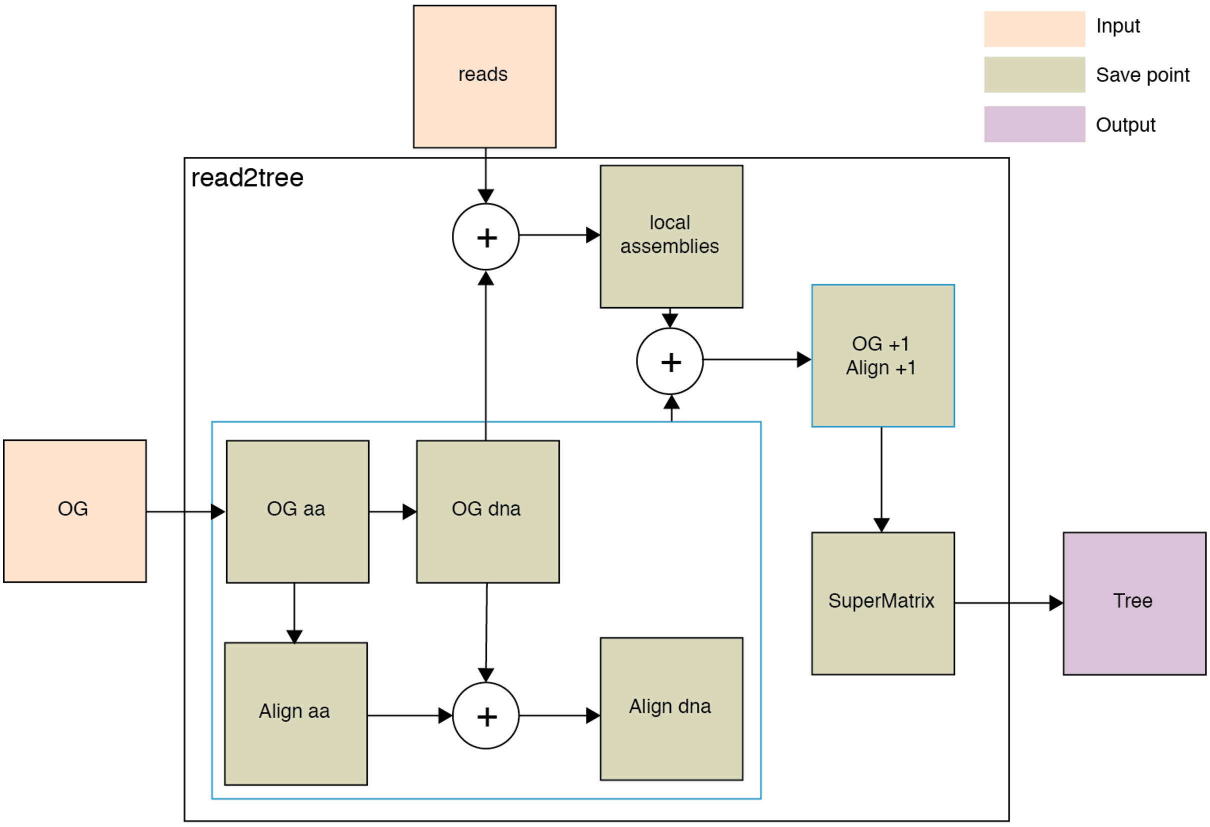

Supplementary Figure 1. Graphical representation of pipeline. All boxes in green are stored by Read2Tree. Inputs are reads and a set of reference orthologous groups that can be selected from over 2000 species from the OMA database. Local assemblies here as reconstructed sequences using the bases placed against the reference.

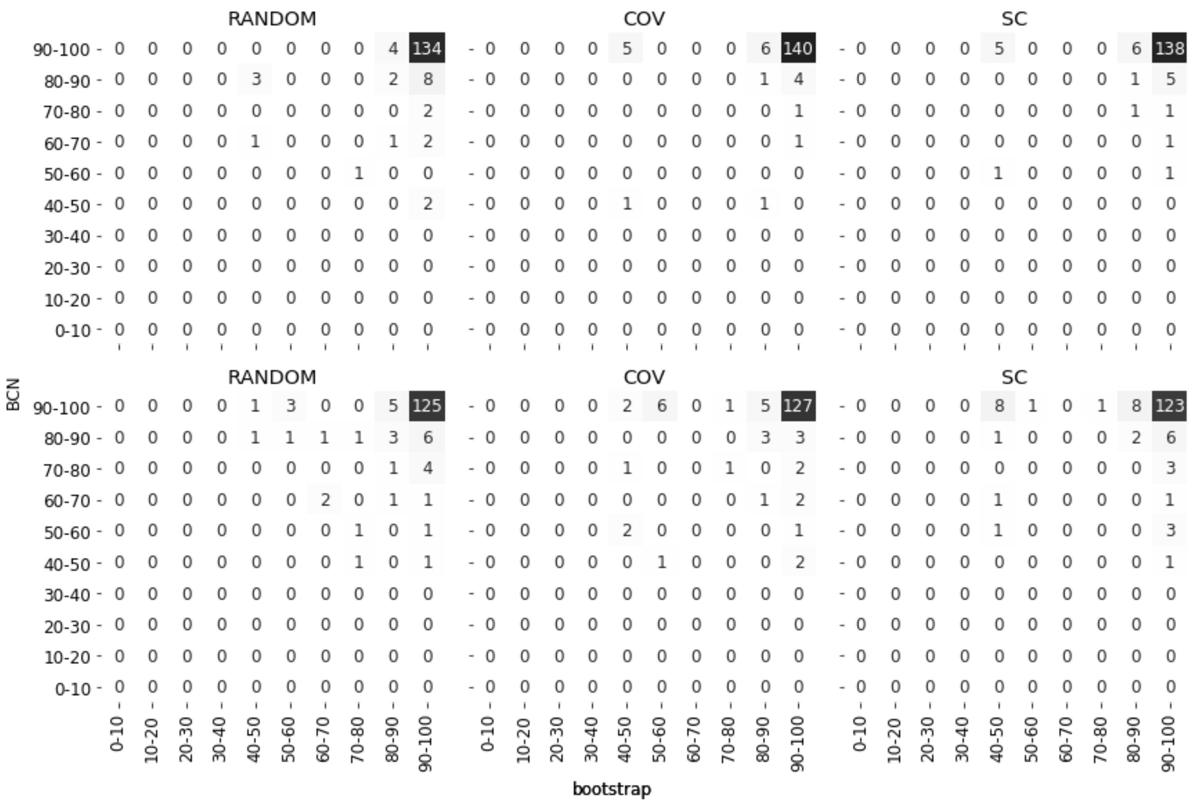

Supplementary Figure 2. Comparison of different gene selection methods in 20 times 8out/8in test show differences in relation between bootstrap on nodes of mapped species and its jaccard similarity with reference tree (Best Corresponding Node BCN) value. Top row shows values obtained using simulated reads and bottom row shows values obtained using real reads. In both cases we see that using coverage as a selection method shows the highest number of nodes where mapped species have high bootstrap and high BCN values.

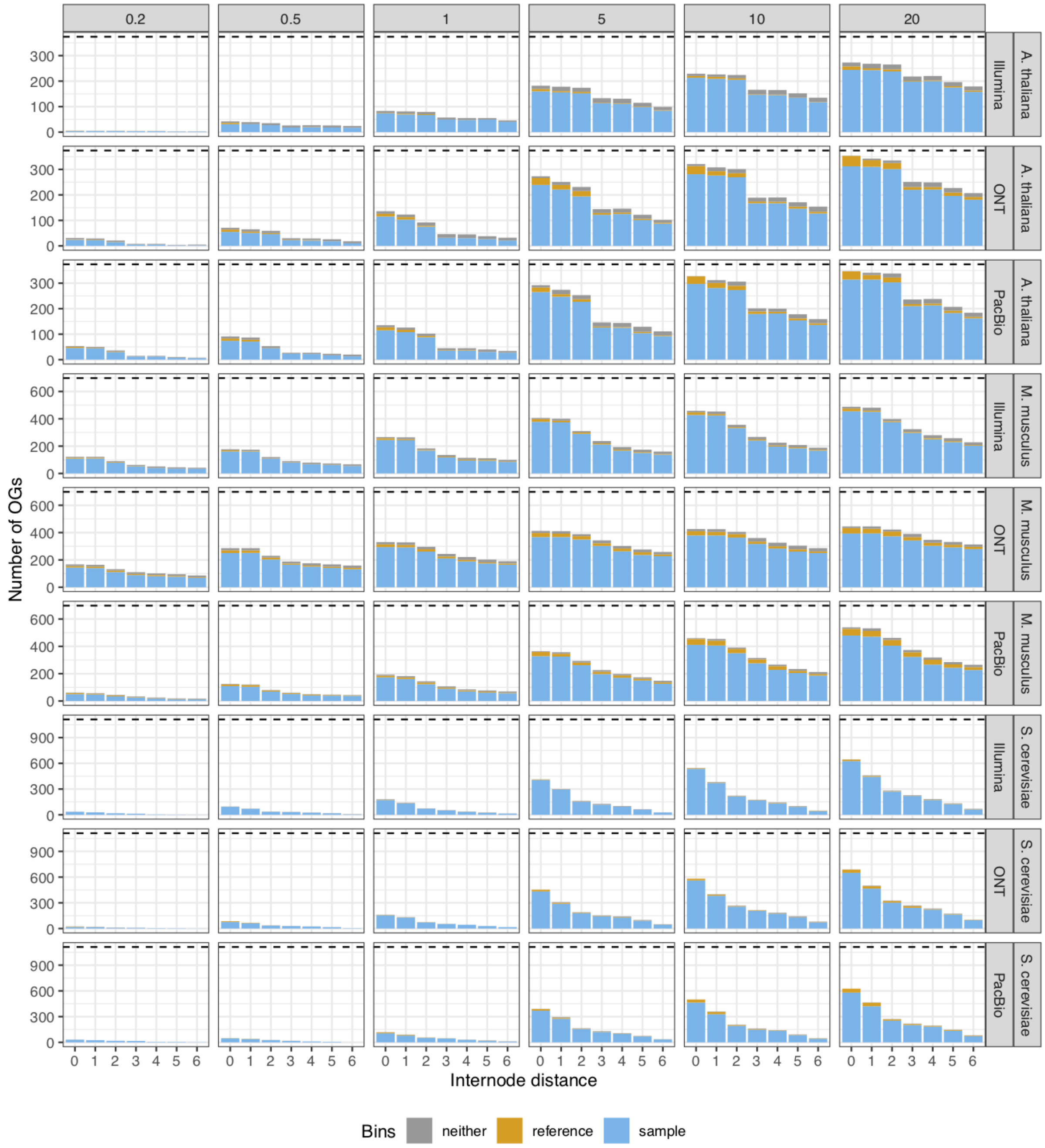

Supplementary Figure 3. Binning of top BlastP results of r2t-sequence of selected species against their original OG (including the removed species) in either being most similar to its assembled counterpart (blue), to its reference used for reconstruction (yellow) or to any other sequence (grey). Results show that Read2Tree if reconstructing a sequence in most cases reconstructs a sequence that shows highest similarity to its assembled counterpart although this sequence was not present in the reference dataset when running Read2Tree.

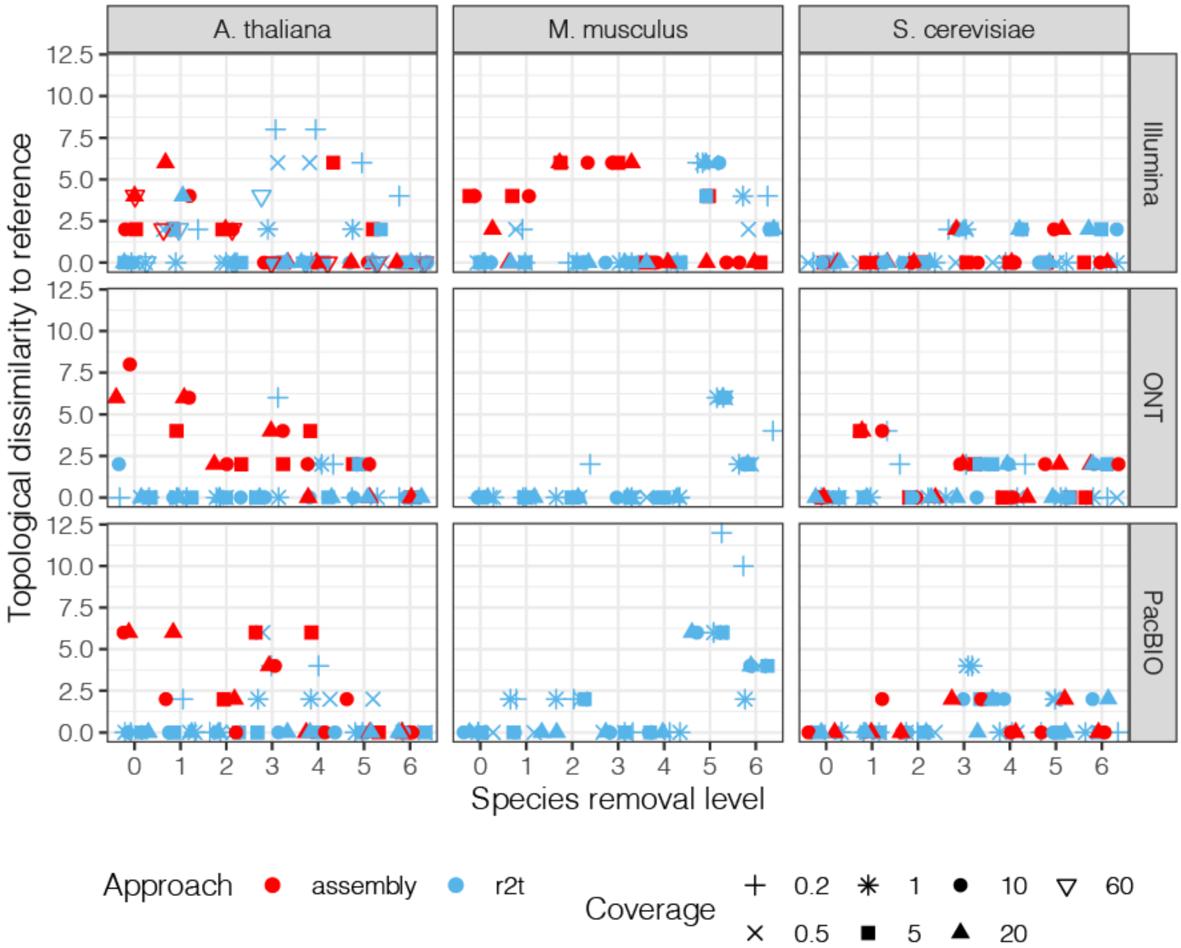

Supplementary Figure 4. Comparison of Robinson Foulds tree distance of Read2Tree against reference tree and assembly obtained tree against reference tree. Read2Tree shows similar performance across technologies, coverage levels and distance to the closest remaining ancestor.

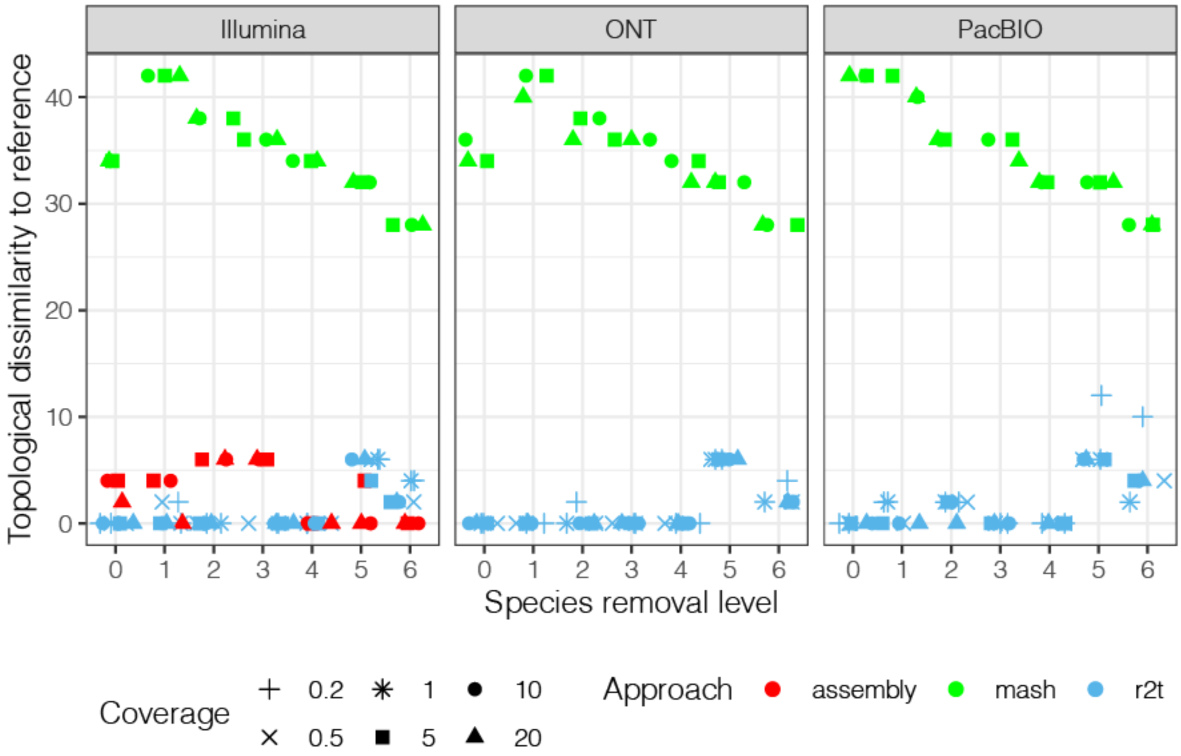

Supplementary Figure 5. Topological dissimilarity (Robinson-Foulds distance) in comparison to reference trees for *M. musculus*. For the mash trees we have downloaded the genome assembly of species from NCBI Assembly. The Mash sketch with the size of 10m was used to create the k-mer sketch (k=21 as default) which was followed by mash distance to calculate the distances between genomes. Finally, RapidNJ was used on the distance matrix to infer the species tree. All trees are provided in Newick format in **Supplementary File 2.**

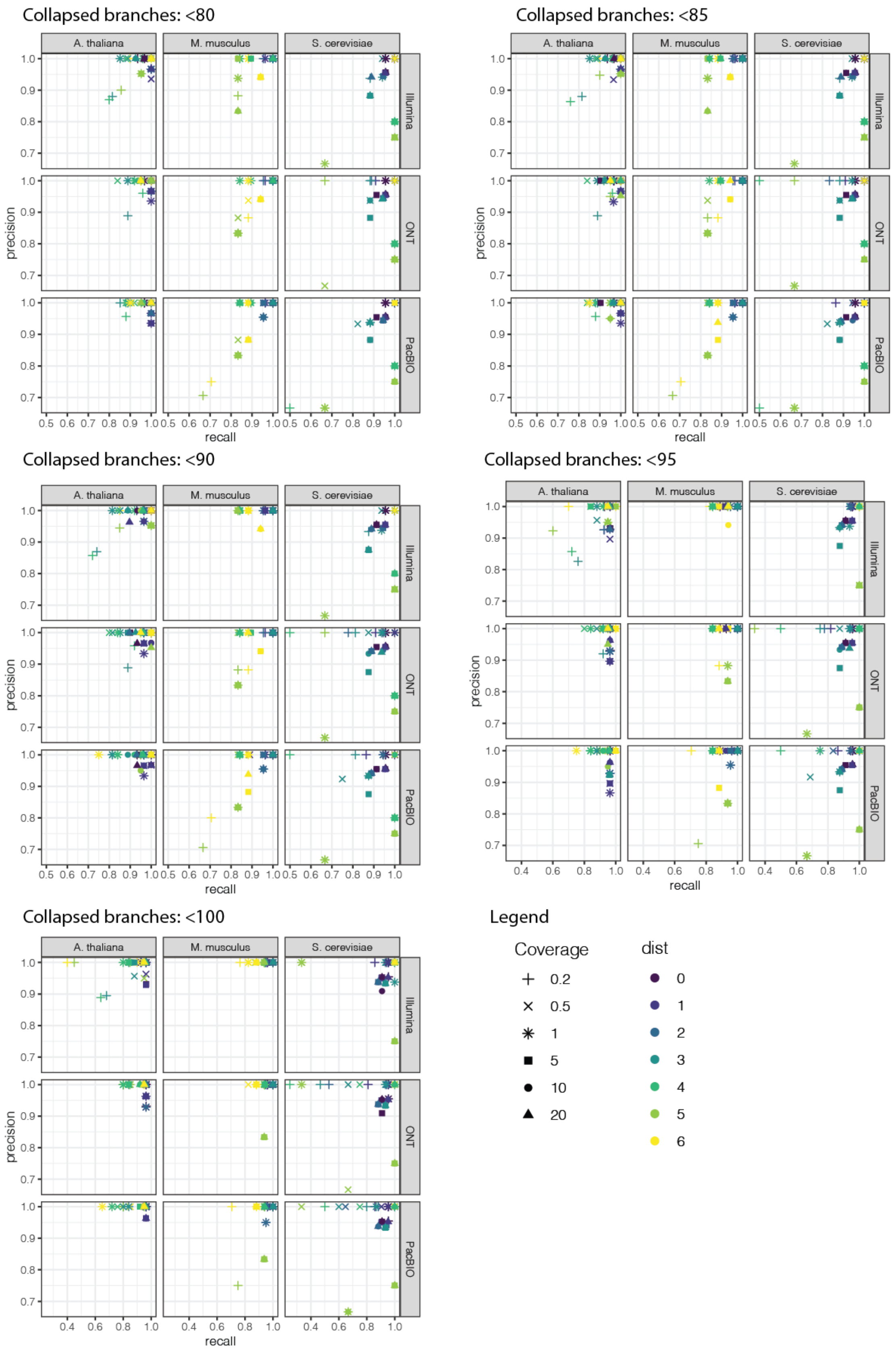

Supplementary Figure 6. Precision recall plots for different levels of bootstraps for which branches were removed. Precision and recall increase for higher levels of bootstrap thresholds.

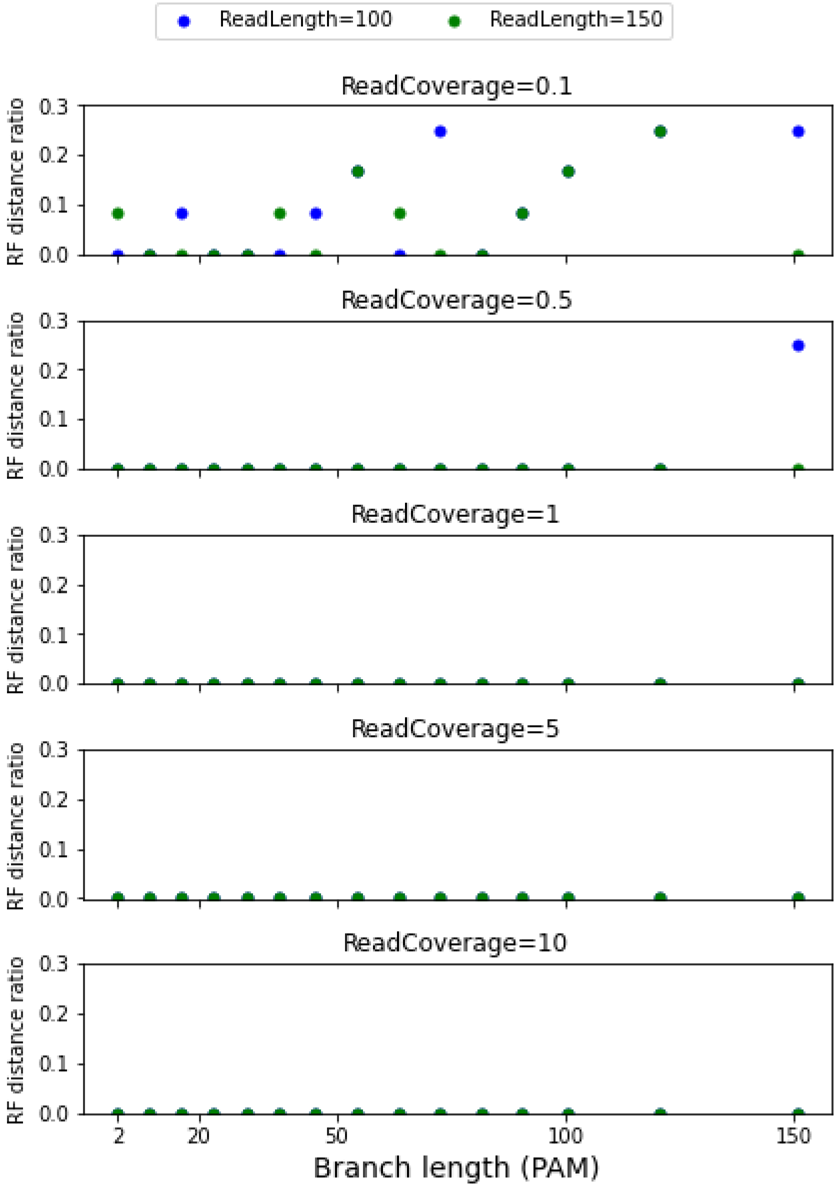

Supplementary Figure 7. Comparison of Robinson Foulds tree distance of Read2Tree against true tree using simulated dataset. The design includes a fixed topology for species tree with 15 species using the ALF package. We varied the branch length leading to one of the species (species of interest) between 2 PAM and 150 PAM. For each run, we infer afterwards the OMA Groups (excluding the species of interest). Then using art_illumina, we generated DNA sequencing reads (paired-end) with length of 100 and 150 and coverage of 0.1 to 10. Next, for each case, we ran the read2tree package to infer the phylogeny. Finally, we calculated the Robinson–Foulds metric between inferred species tree and the true one (output of ALF). As one can see from the figure, only in 0.1x coverage inferring the true tree is challenging, otherwise in almost all the cases the tree is inferred perfectly.

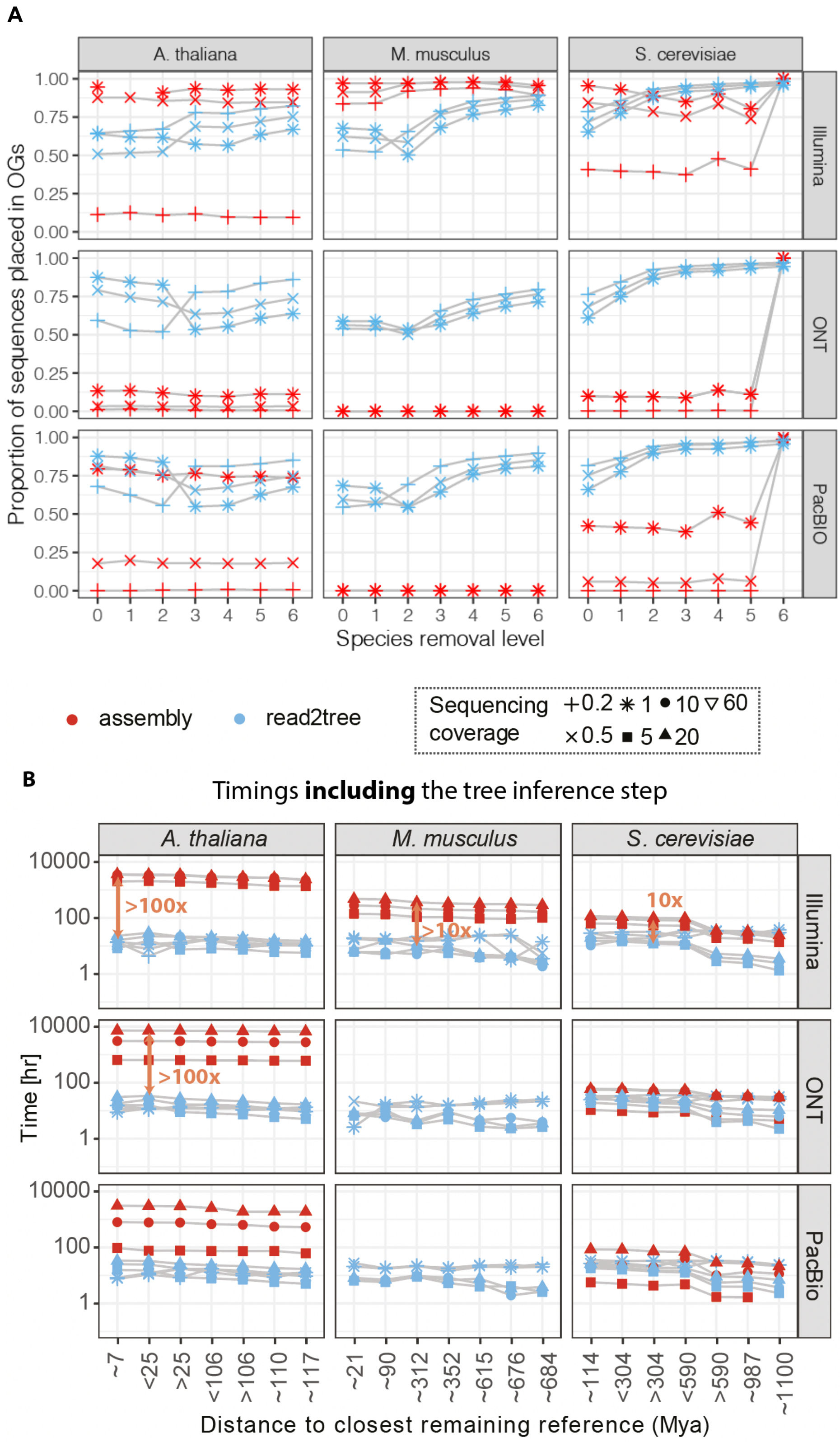

Supplementary Figure 8. Additional details on analysis of Figure 3 from the main text. **A**. Proportions of sequences placed into the total number of OGs when selecting OGs with at least 80% taxa. **B**. CPU time required *including* the tree inference step.

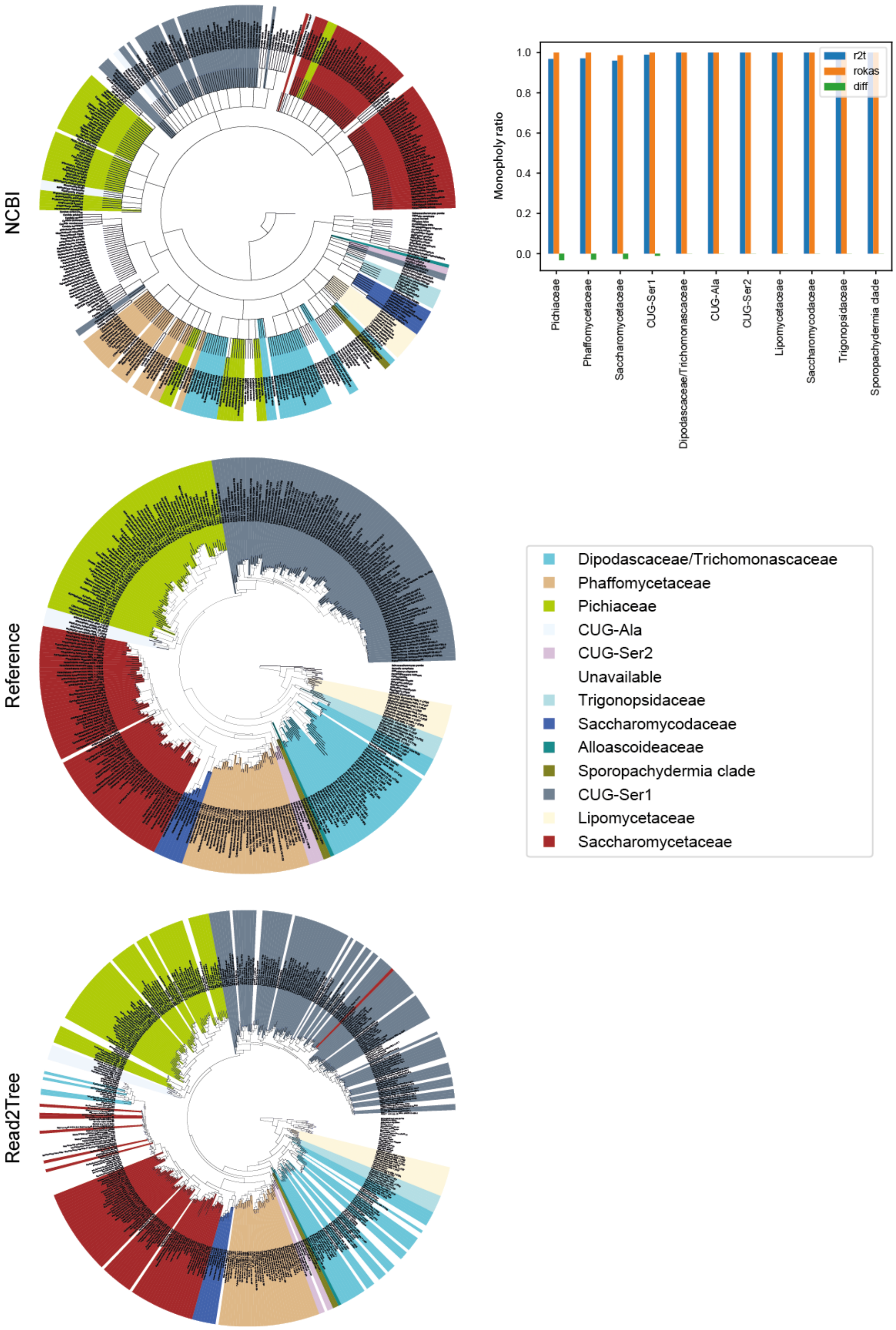

Supplementary Figure 9. Comparison of Trees by classification based on (Shen et al., 2018) at order level. Left NCBI, reference and Read2Tree inferred trees. Right Monophyly score computed for Shen *et al.* classification. Read2Tree is nearly as precise and the standard pipeline in recapitulating the right monophyletic groupings.

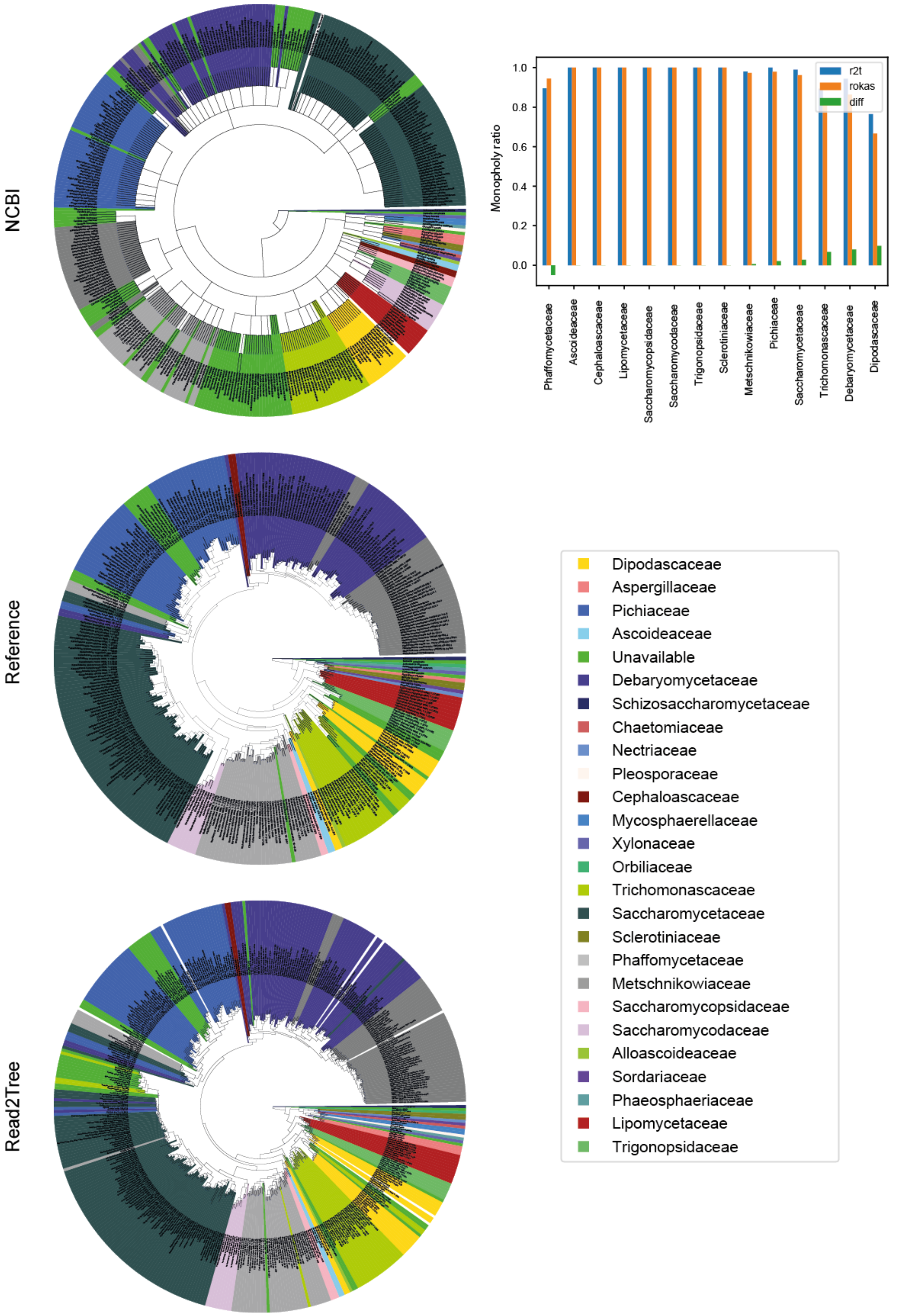

Supplementary Figure 10. Comparison of Trees by classification based on (Shen et al., 2018) at family level. Left NCBI, reference and Read2Tree inferred trees. Right Monophyly score computed for Shen *et al.* classification. Read2Tree is nearly as precise and the

standard pipeline in recapitulating the right monophyletic groupings. Similar comparison at order level is provided in Supplementary Figure 9.

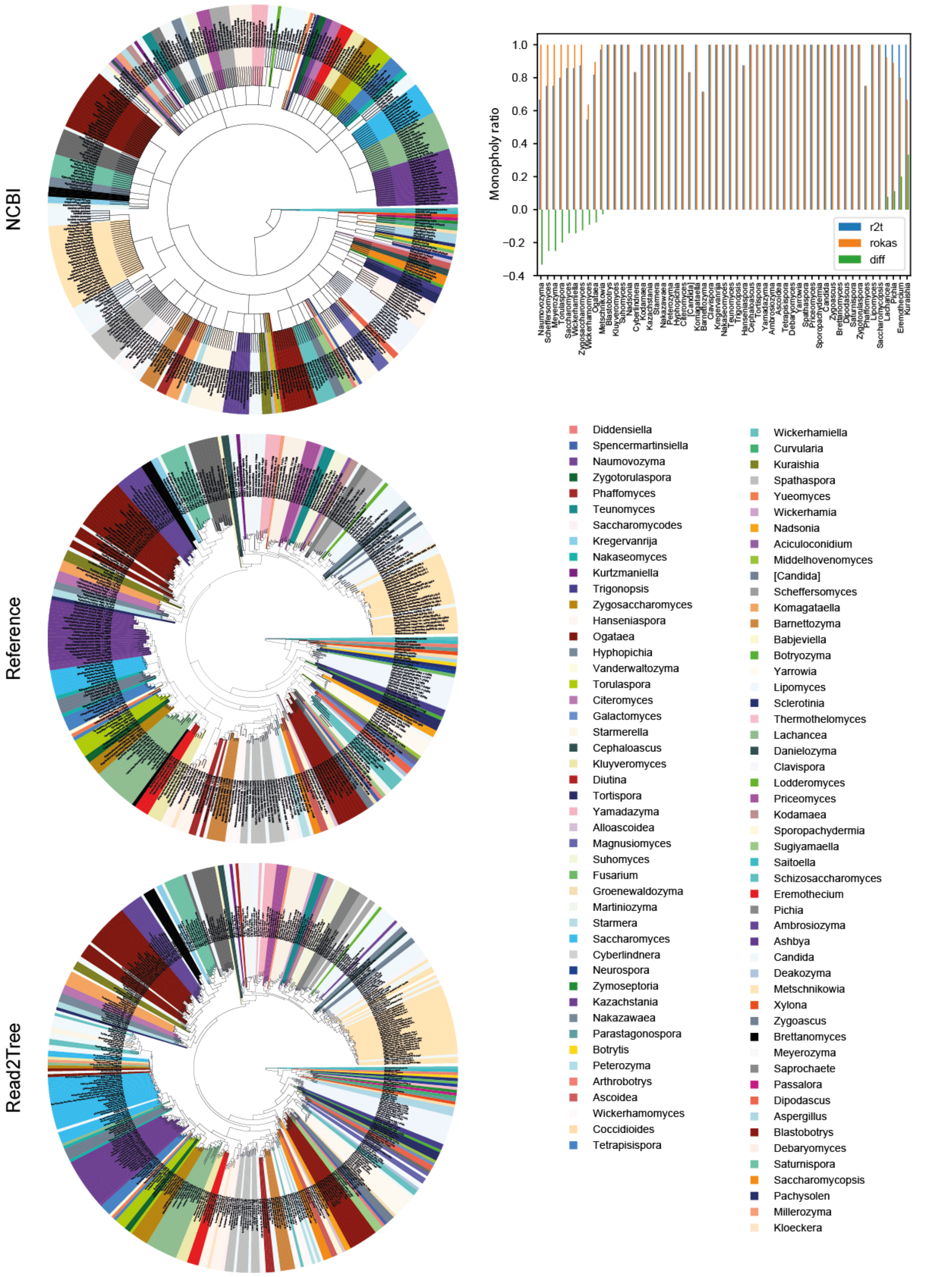

Supplementary Figure 11. Comparison of Trees by classification based on (Shen et al., 2018) at genus level. Left NCBI, reference and Read2Tree inferred trees. Right Monophyly score computed for Shen *et al.* classification. Read2Tree is nearly as precise and the standard pipeline in recapitulating the right monophyletic groupings.

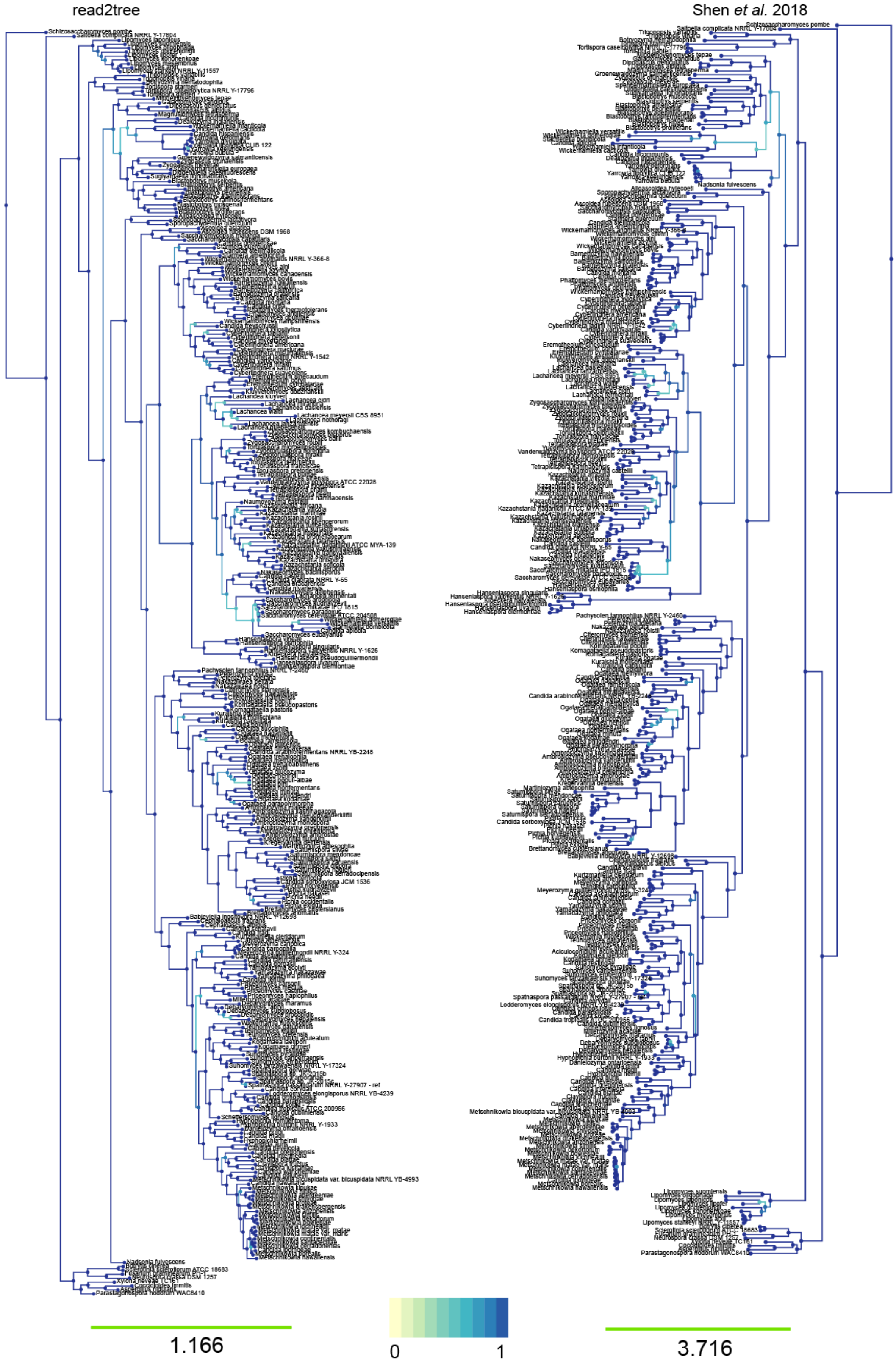

Supplementary Figure 12. Side by side comparison of read2tree using phylo.io. In dark blue are all the branches that are equal between the two trees using the best corresponding node algorithm.

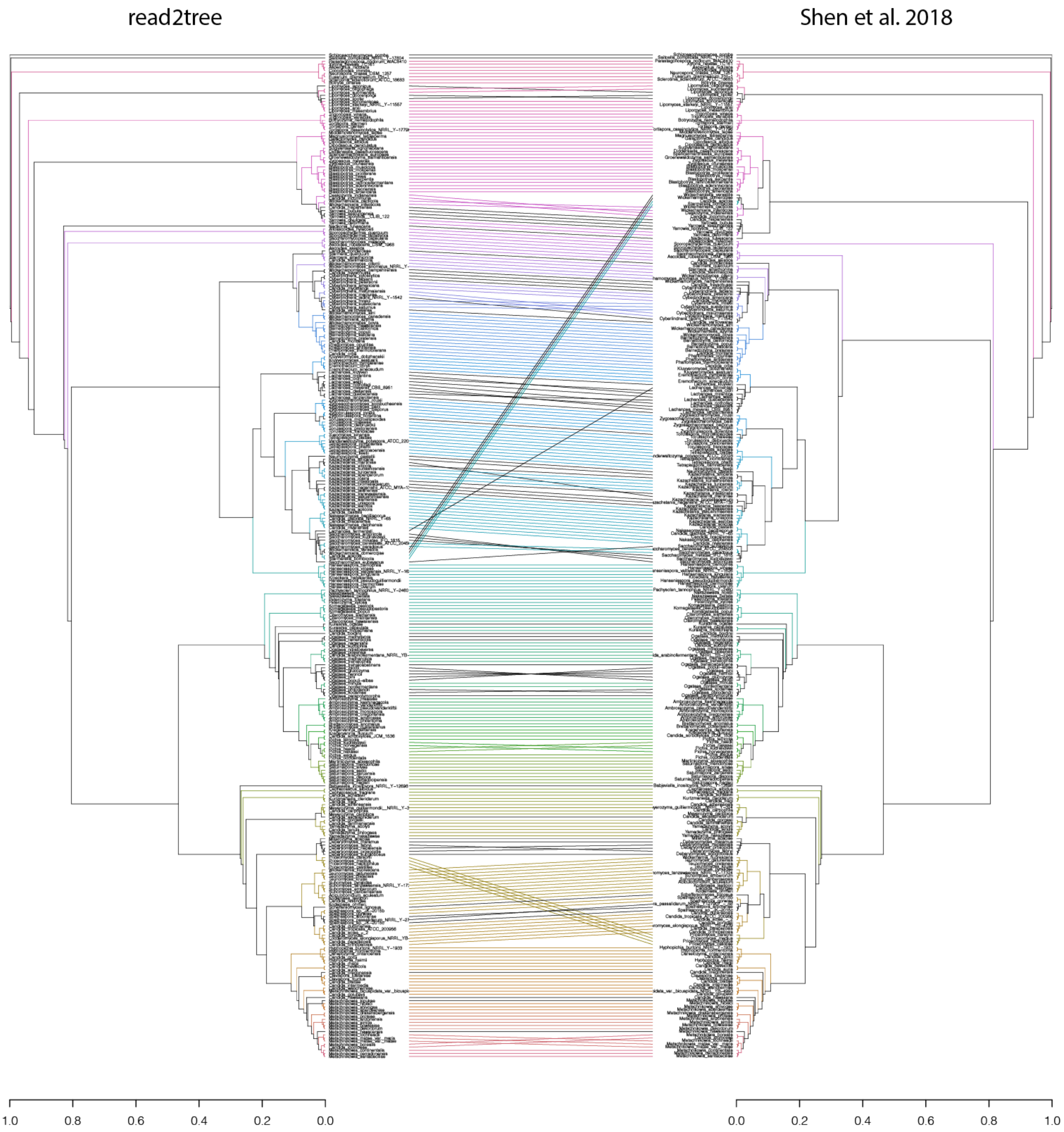

Supplementary Figure 13 . Tanglegram of yeast tree comparison highlighting differences and similarities in the tree. Entanglement coefficient was 0.0096. Tanglegram was produced using the dendextend R library.

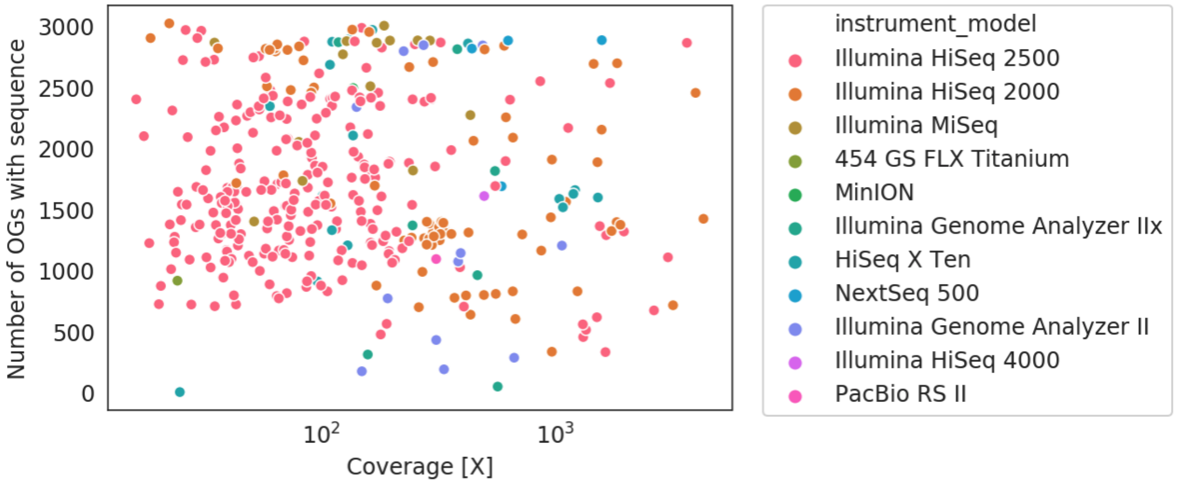

Supplementary Figure 14 . Coverage to the number of obtained sequence relationships. Even for low coverage we see a large number of obtained sequences. No clear relationship is present between input coverage and number of sequences placed in OGs. In purple at 100X we display the number of sequences present in all ~3000 OGs for the reference species.

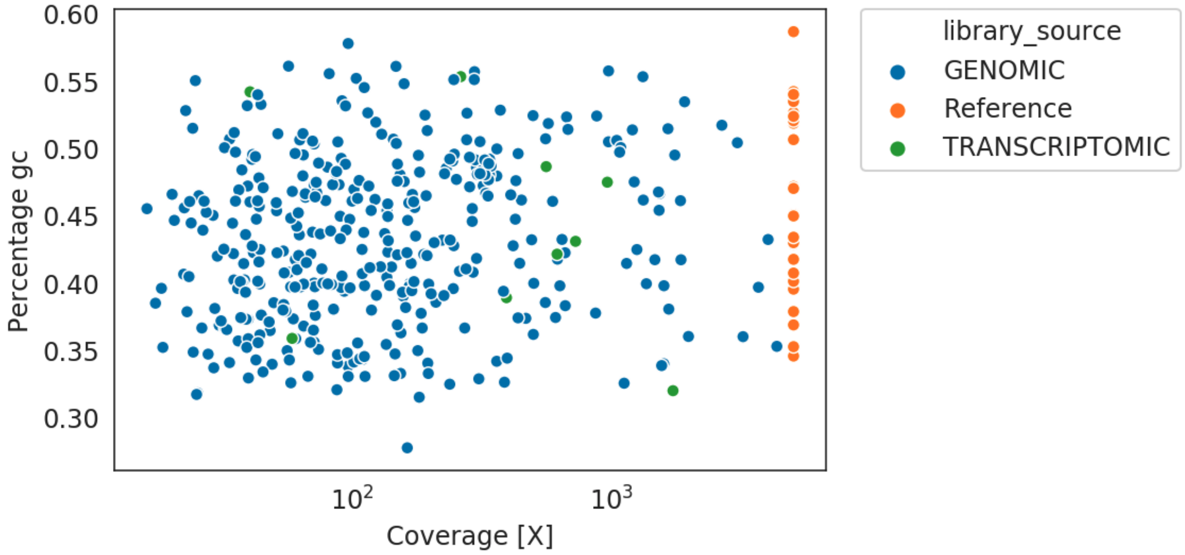

Supplementary Figure 15. Average percentage GC content in obtained sequences for yeast tree obtained using Read2Tree in comparison to given references. In most cases the reconstructed sequences are within the range of GC content as present in the reference sequences. References sequence artificially set to 5000X coverage for display purposes.

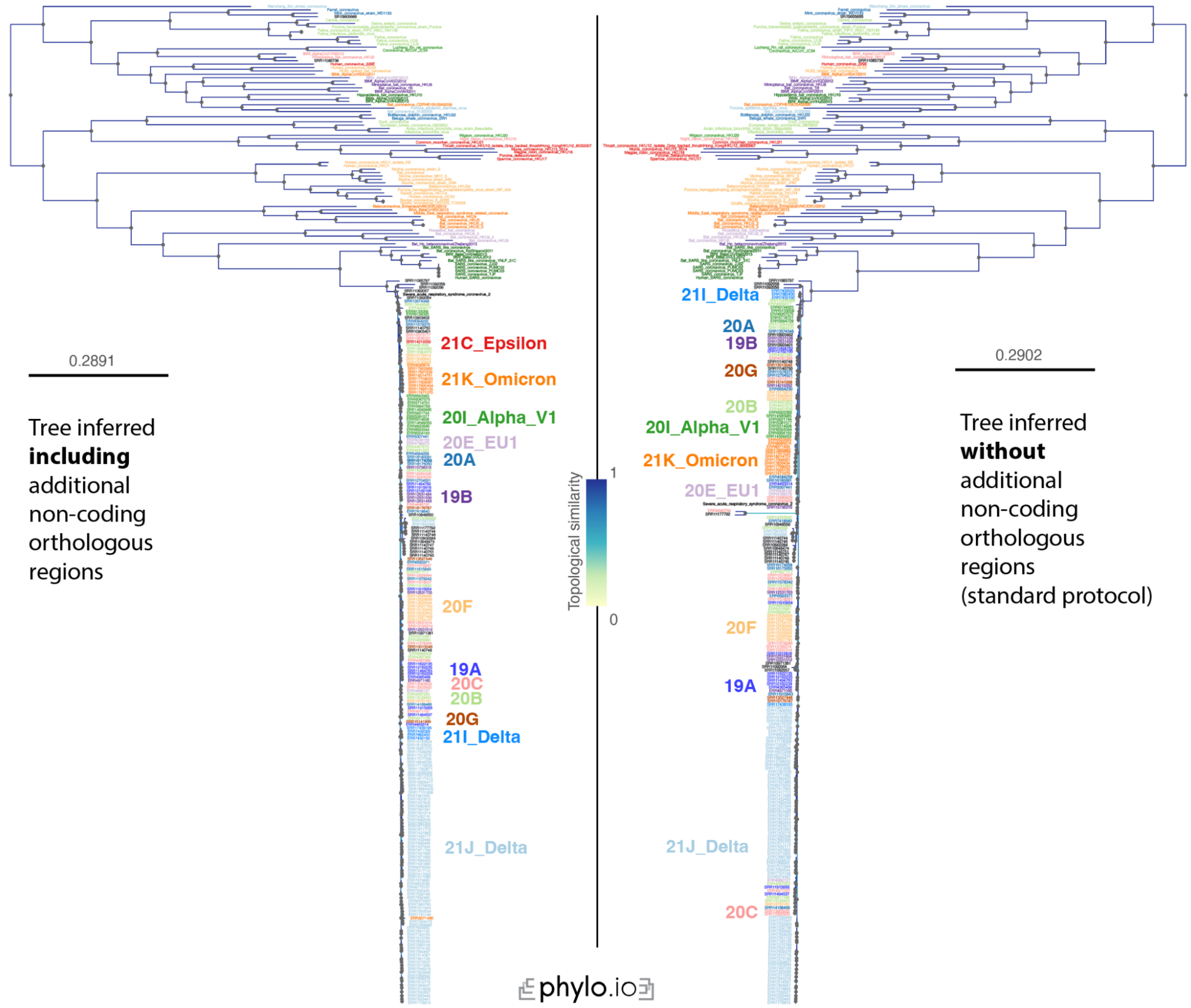

Supplementary Figure 16. Comparison of Read2Tree-reconstructed Coronaviridae trees with (left) and without (right) additional non-coding reference regions (see Methods). The backbone is 100% consistent, while the SARS-CoV-2 part of the tree shows a bit more variation. Nevertheless, the colour clustering in both trees shows that Read2Tree can be used to reliably classify samples according to the fine-grained Nextstrain (Hadfield et al., 2018) classification. Best performance is obtained when including additional non-coding reference markers as described in the *Methods* section.

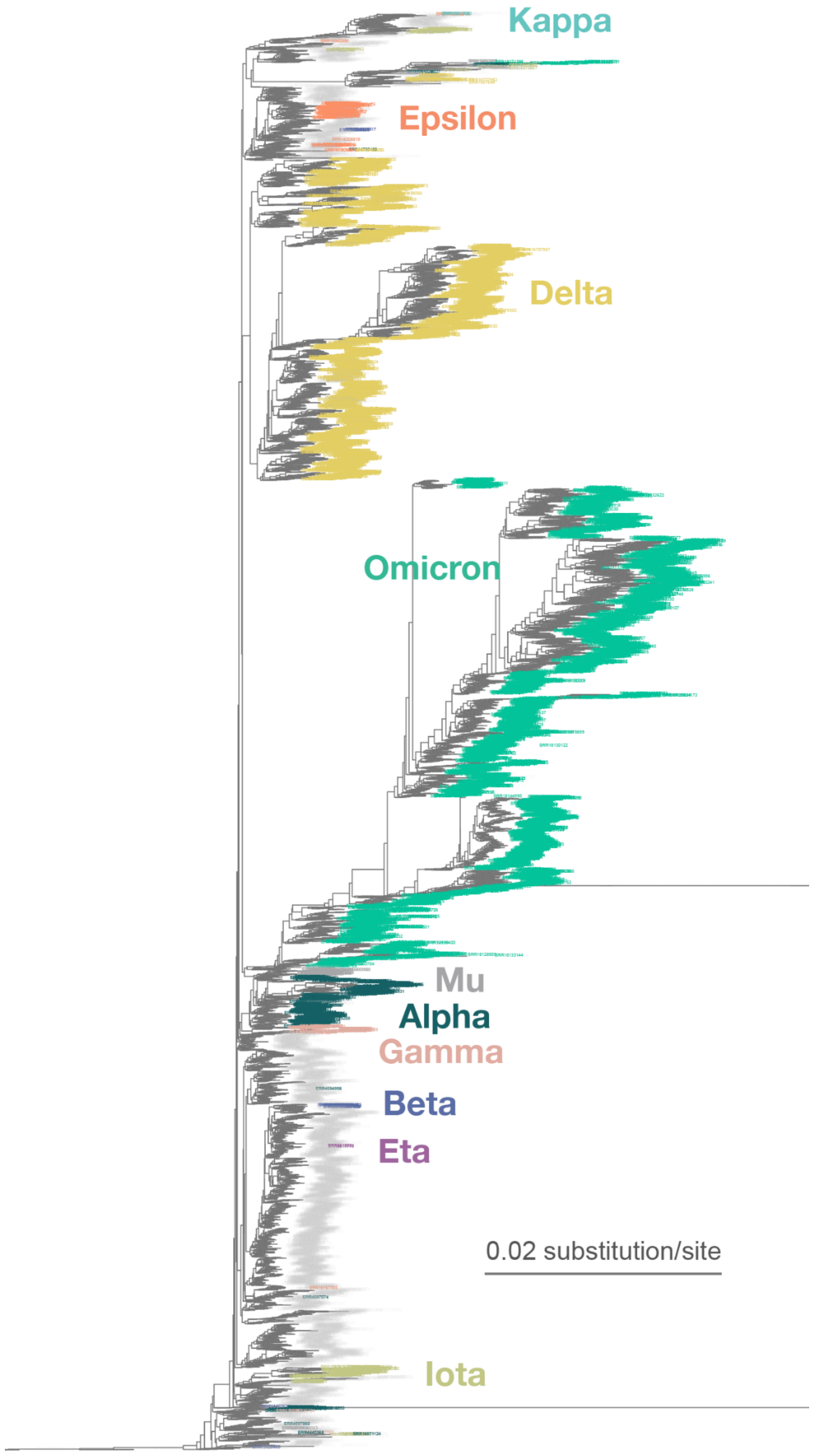

Supplementary Figure 17. Zoomed-in display of a tree inferred using Read2Tree on 10,283 samples whole genome SARS-CoV-2 samples. Classification in colour was obtained from https://harvestvariants.info/ (see Methods), where grey leaves are unclassified according to the CDC label. The colour clustering shows that the Read2Tree-based tree recovers consistent classification.

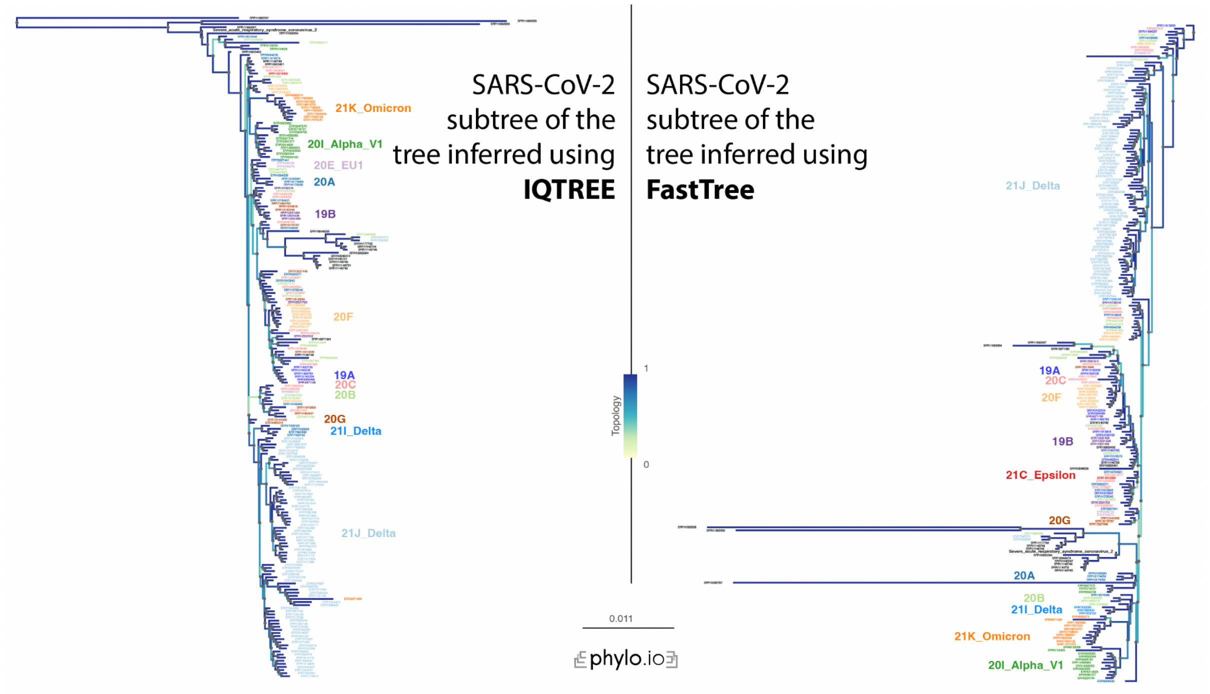

Supplementary Figure 18. Comparison of IQTree and FastTree for Coronaviridae. Classification can be done quite reliably with both tree inference methods, but the rooting appears to be incorrect in the FastTree-reconstructed tree. The two trees are provided in the Supplementary File 2 in Newick format.
