# Supplementary material for: Inference of phylogenetic trees directly from raw sequencing reads using Read2Tree
Source: Nat Biotechnol. 2023 Apr 20;42(1):139–47. doi: 10.1038/s41587-023-01753-4 (PMC10791578; doi:10.1038/s41587-023-01753-4)
Supplement: Supplementary file 2 — Reporting Summary [file 41587_2023_1753_MOESM2_ESM.pdf]

nature research

Corresponding author(s): Fritz Sedlazeck, Christophe Dessimoz

Last updated by author(s): Feb 17, 2023

# Reporting Summary

Nature Research wishes to improve the reproducibility of the work that we publish. This form provides structure for consistency and transparency in reporting. For further information on Nature Research policies, see our Editorial Policies and the Editorial Policy Checklist.

## Statistics

For all statistical analyses, confirm that the following items are present in the figure legend, table legend, main text, or Methods section.

| n/a | Confirmed | |
|---|---|---|
| ☐ | ☒ | The exact sample size (*n*) for each experimental group/condition, given as a discrete number and unit of measurement |
| ☒ | ☐ | A statement on whether measurements were taken from distinct samples or whether the same sample was measured repeatedly |
| ☒ | ☐ | The statistical test(s) used AND whether they are one- or two-sided<br>*Only common tests should be described solely by name; describe more complex techniques in the Methods section.* |
| ☒ | ☐ | A description of all covariates tested |
| ☒ | ☐ | A description of any assumptions or corrections, such as tests of normality and adjustment for multiple comparisons |
| ☐ | ☒ | A full description of the statistical parameters including central tendency (e.g. means) or other basic estimates (e.g. regression coefficient) AND variation (e.g. standard deviation) or associated estimates of uncertainty (e.g. confidence intervals) |
| ☒ | ☐ | For null hypothesis testing, the test statistic (e.g. *F*, *t*, *r*) with confidence intervals, effect sizes, degrees of freedom and *P* value noted<br>*Give P values as exact values whenever suitable.* |
| ☒ | ☐ | For Bayesian analysis, information on the choice of priors and Markov chain Monte Carlo settings |
| ☒ | ☐ | For hierarchical and complex designs, identification of the appropriate level for tests and full reporting of outcomes |
| ☒ | ☐ | Estimates of effect sizes (e.g. Cohen's *d*, Pearson's *r*), indicating how they were calculated |

*Our web collection on statistics for biologists contains articles on many of the points above.*

## Software and code

Policy information about availability of computer code

| | |
|---|---|
| Data collection | Data used for this study was obtained from the NCBI Short Read Archive database and from the OMA orthology browser. |

| | |
|---|---|
| Data analysis | Data analysis was performed using python jupyter notebooks and R markdowns and are available in a github repository (https://github.com/dvdylus/read2tree_paper). Code for main method presented in this paper is available here: https://github.com/DessimozLab/read2tree.<br><br>MAFFT v7.310 (--maxiter 1000 --local)<br>IQTREE v1.6.9 ( -m LG -nt 4 -mem 4G -seed 12345 -bb 1000)<br>blastp (ncbi-blast; v2.8.1)<br>megahit (v1.2.9) with default parameters<br>SOAPdenovo (version 2.04-r241) for scaffolding: First, SOAPdenovo-fusion -D -K 41 -c megahit.contigs.fa -g scaffold_prefix -p 20 followed by SOAPdenovo-mer map and scaff with recommended parameters over the config file. For ONT reads we assembled the reads using Canu (v2.0) with a specified genome size (genomeSize)<br>PacBio CLR data we also used Canu (v2.0) with similar parameters, but specifying the -pacbio-raw parameter<br>For Illumina RNA seq, we used Trinity (v2.8.5) with the following parameters: --seqType fq --max_memory 50G --left reads1.fq.gz --right reads2.fq.gz --CPU 6 --trimmomatic --full_cleanup --output prefix<br>OMA standalone (v2.3.3) with default parameters<br>iss (v1.3.0 https://github.com/HadrienG/InSilicoSeq, --model hiseq -n 600000)<br>phyutils 2.2.6 (seqs -aa -clean 0.01)<br>trimAl v1.4.rev15 (-gappyout)<br>For the COVID tree: IQTree2 (version 2.2.0-beta) with parameters -m GTR -ninit 2 -me 0.05, as well as FastTree version 2.1.11 as control<br>MASH (version 2.3) sketch with a size of 10m (k=21 as default)<br>RapidNJ (version 2.3.2) |

For manuscripts utilizing custom algorithms or software that are central to the research but not yet described in published literature, software must be made available to editors and reviewers. We strongly encourage code deposition in a community repository (e.g. GitHub). See the Nature Research guidelines for submitting code & software for further information.

## Data

Policy information about availability of data

All manuscripts must include a data availability statement. This statement should provide the following information, where applicable:
- Accession codes, unique identifiers, or web links for publicly available datasets
- A list of figures that have associated raw data
- A description of any restrictions on data availability

Reference species and their sequences are displayed in Figure 2 and are present in the supplement. The exact references used for all the results obtained are also available in a GitHub repository (https://github.com/dvdylus/read2tree_paper). All SRA identifiers are present in Supplementary File 1.

All accession codes are available in the supplement of the paper. Supplement, reference datasets for initial benchmark are deposited here: https://github.com/dvdylus/read2tree_paper. Reference datasets can also be obtained directly from the OMA browser as described in the methods.

# Field-specific reporting

Please select the one below that is the best fit for your research. If you are not sure, read the appropriate sections before making your selection.

☒ Life sciences ☐ Behavioural & social sciences ☐ Ecological, evolutionary & environmental sciences

For a reference copy of the document with all sections, see nature.com/documents/nr-reporting-summary-flat.pdf

# Life sciences study design

All studies must disclose on these points even when the disclosure is negative.

| | |
|---|---|
| Sample size | The budding yeast study size was based on data available in the Short Read Archive at the time we compiled the dataset. For the SARS-CoV-2 sequences, we identified the subset of samples from the open Nextstrain built that had available reads in the Short Read Archive or the European Read Archive. |
| Data exclusions | For the budding yeast, species with coverage below 1X were excluded (this criterion was pre-established). No dataset was excluded from the SARS-CoV-2 dataset. |
| Replication | The key results of the work (namely the speed and accuracy of Read2Tree was replicated on several disjoint datasets, including plants, fungi, vertebrate, coronaviruses) as well as simulation. |
| Randomization | As phylogenetic inference seeks to reconstruct events that happen deep in the past, no randomisation is typically possible. However, we used a variety of data, including simulated data for which all sources of variation can be controlled. Furthermore, the risk of confounders was minimised by adhering to commonly accepted standards for phylogenetic studies. |
| Blinding | As this was not a randomised controlled study, blinding is not relevant. |

# Reporting for specific materials, systems and methods

We require information from authors about some types of materials, experimental systems and methods used in many studies. Here, indicate whether each material, system or method listed is relevant to your study. If you are not sure if a list item applies to your research, read the appropriate section before selecting a response.

## Materials & experimental systems

| n/a | Involved in the study |
|---|---|
| ☒ | ☐ Antibodies |
| ☒ | ☐ Eukaryotic cell lines |
| ☒ | ☐ Palaeontology and archaeology |
| ☒ | ☐ Animals and other organisms |
| ☒ | ☐ Human research participants |
| ☒ | ☐ Clinical data |
| ☒ | ☐ Dual use research of concern |

## Methods

| n/a | Involved in the study |
|---|---|
| ☒ | ☐ ChIP-seq |
| ☒ | ☐ Flow cytometry |
| ☒ | ☐ MRI-based neuroimaging |
